# A molecular odorant transduction model and the complexity of spatio-temporal encoding in the *Drosophila* antenna

**DOI:** 10.1371/journal.pcbi.1007751

**Published:** 2020-04-14

**Authors:** Aurel A. Lazar, Chung-Heng Yeh

**Affiliations:** Department of Electrical Engineering, Columbia University, New York, New York, United States of America; University of California Davis Department of Plant Sciences, UNITED STATES

## Abstract

Over the past two decades, substantial amount of work has been conducted to characterize different odorant receptors, neuroanatomy and odorant response properties of the early olfactory system of *Drosophila melanogaster*. Yet many odorant receptors remain only partially characterized, and the odorant transduction process and the axon hillock spiking mechanism of the olfactory sensory neurons (OSNs) have yet to be fully determined. Identity and concentration, two key characteristics of the space of odorants, are encoded by the odorant transduction process. Detailed molecular models of the odorant transduction process are, however, scarce for fruit flies. To address these challenges we advance a comprehensive model of fruit fly OSNs as a cascade consisting of an odorant transduction process (OTP) and a biophysical spike generator (BSG). We model odorant identity and concentration using an odorant-receptor binding rate tensor, modulated by the odorant concentration profile, and an odorant-receptor dissociation rate tensor, and quantitatively describe the mechanics of the molecular ligand binding/dissociation of the OTP. We model the BSG as a Connor-Stevens point neuron. The resulting spatio-temporal encoding model of the *Drosophila* antenna provides a theoretical foundation for understanding the neural code of both odorant identity and odorant concentration and advances the state-of-the-art in a number of ways. First, it quantifies on the molecular level the spatio-temporal level of complexity of the transformation taking place in the antennae. The concentration-dependent spatio-temporal code at the output of the antenna circuits determines the level of complexity of olfactory processing in the downstream neuropils, such as odorant recognition and olfactory associative learning. Second, the model is biologically validated using multiple electrophysiological recordings. Third, the model demonstrates that the currently available data for odorant-receptor responses only enable the estimation of the affinity of the odorant-receptor pairs. The odorant-dissociation rate is only available for a few odorant-receptor pairs. Finally, our model calls for new experiments for massively identifying the odorant-receptor dissociation rates of relevance to flies.

## Author summary

Identity and concentration, intrinsically embedded in the odorant space, are two key characteristics of olfactory coding that define the level of complexity of neural processing throughout the olfactory system in the fruit fly. In this paper we advance a theoretical foundation for understanding these two characteristics by quantifying mathematically the odorant space and devising a biophysical model of the olfactory sensory neurons (OSNs). To validate our modeling approach, we propose and apply an algorithm to estimate the affinity value and the dissociation rate, the two characteristics that define odorant identity, of multiple odorant-receptor pairs. We then evaluate our model with a multitude of odorant waveforms and demonstrate that the model output reproduces the temporal responses of OSNs obtained from in vivo electrophysiology recordings. Furthermore, we evaluate the model at the OSN population level and quantify on the molecular level the spatio-temporal level of complexity of the transformation taking place between the odorant space and the OSNs. The resulting concentration-dependent spatio-temporal code determines the level of complexity of the input space driving olfactory processing in the downstream neuropils. Lastly, our model demonstrates that the currently available data for OSN responses only enables estimation of affinity value. This calls for new experiments for massively identifying the odorant-receptor dissociation rates of relevance to flies.

## Introduction

The odorant response of olfactory sensory neurons in the *Drosophila* antennae has been experimentally characterized by multiple research groups [1–3], and their results have been combined into a single consensus database, called the DoOR database [4, 5]. A key functionality of OSNs is to jointly encode both odorant identity and odorant concentration [6–10].

A single odorant stimulus usually activates multiple OSN groups that express the same receptor type; likewise different odorants activate different OSN groups [11–13]. Such an OSN coding scheme is universal in insects and vertebrates, and has been called a “combinatorial odorant code” in [14]. For added precision, we characterize the odorant code as “spatio-temporal”. The identity of an odorant, mono-molecular odorant or mixture alike, is encoded by the combination (i.e., vector) of responding OSN groups [1, 2], and each OSN group serves as one vector component of the code for a set of odorants. For each odorant, the size of the set of the actively spiking OSNs varies as the concentration amplitude changes [2].

The two key characteristics of the odorant space, identity and concentration, are encoded by the odorant transduction process [8, 15] taking place in the cilia of the OSNs. However, detailed molecular models of the odorant transduction process are scarce for fruit flies. The spike train generated by an OSN simultaneously encodes the odorant concentration and concentration gradient, also known as 2D odorant encoding [16, 17].

To address these challenges we advance a comprehensive model of fruit fly OSNs as a cascade consisting of an odorant transduction process (OTP) and a biophysical spike generator (BSG). We model identity and concentration in OTP by an odorant-receptor binding rate tensor, modulated by the odorant concentration profile, and an odorant-receptor dissociation rate tensor, and quantitatively describe the ligand binding/ dissociation process. The three entities, the concentration profile, the binding rate tensor, and the dissociation rate tensor, jointly determine the level of complexity of the odorant space, and thereby, the level of complexity of the spatio-temporal encoding circuits.

OSNs are distributed across the surface of maxillary palp and the third segment of antenna [1, 18, 19]. Since there is no commonly accepted terminology in the literature for naming these two olfactory appendages as a single entity, and in order to avoid potential confusion, we will refer to the set of all OSNs on one side of the fly brain as an antenna/maxillary palp (AMP) local processing unit (LPU) [20].

To biologically validate our modeling approach, we first propose an algorithm for estimating the affinity and the dissociation rate of an odorant-receptor pair. The affinity is defined as the ratio between the binding rate and the dissociation rate. We then apply the algorithm to electrophysiology recordings and estimate the affinity and dissociation rate for three odorant-receptor pairs. Second, we evaluate the temporal response of the OSN model with a multitude of stimulus waveforms for all three odorant-receptor pairs. The output of the model closely reproduces the temporal responses of OSNs obtained from *in vivo* electrophysiological recordings [16, 17] for all three odorant-receptor pairs across all three types of stimulus waveforms.

Lastly, we evaluate the spatio-temporal encoding model of the OSNs at the population level, i.e., LPU level. We first empirically estimate the odorant-receptor affinity using the spike count records in the DoOR database for 24 receptor types in response to 110 odorants [5]. With estimated affinity values, we simulate the temporal response of the OSN population to staircase odorant waveforms. The output of the simulated OSN population demonstrates that the odorant identity is encoded in the set of odorant-activated OSN groups expressing the same receptor type, and, more importantly, the size of the set expands or reduces as the odorant concentration increases or decreases.

The fruit fly OSN model presented here provides a theoretical foundation for understanding on the molecular level the encoding of both odorant identity and odorant concentration. The odorant space model introduced here enables the development, analysis and validation of odorant transduction and representation in the spike domain by the antenna OSNs, and odor signal processing in the antennal lobe and beyond.

## Results

### The OSN model

Detailed biophysical models for the odorant transduction process have been proposed for worms and vertebrates. Such models are scarce for insects and, in particular, for fruit flies. Dougherty et al. proposed a frog odorant receptor model that exhibits a complex temporal response [21]. Rospars et al. proposed a model that characterizes the steady state response of OSNs for rats and moths [22, 23]. The model stands out for its simplicity and modeling clarity, while lacking temporal variability. Other notable models appeared in [24, 25]. Recently, Cao et al. published a phenomenological model to characterize the peak and the steady response of sensory adaption for fruit fly OSNs [26]. Gorur-Shandilya et al. proposed a two-state model for the fruit fly odorant receptors that can reproduce Weber-Fechner’s law observed in physiological recordings [27]. In addition, De Palo et al. [28] proposed an abstract/phenomenological model with feedback mechanism that characterizes the common dynamical features in both visual and olfactory sensory transduction.

**Fig 1:**
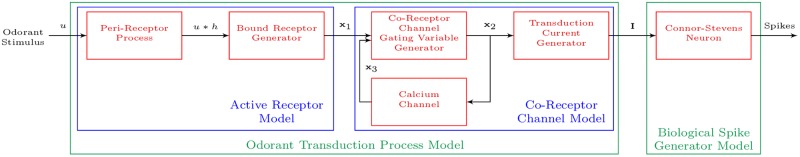
The block diagram of fruit fly OSN model consists of the OTP and the BSG.

Except for the transduction current recorded for studying sensory adaptation [26], modeling the temporal response of the AMP LPU on either side of the brain has been scarcely investigated in the literature. In particular, 2D odorant encoding has not yet been successfully modeled. To address these challenges, we model the OSNs as a cascade consisting of an odorant transduction process (OTP) and a biophysical spike generator (BSG), as shown in Fig 1. The OTP model consists of an active receptor model and a co-receptor channel model [29, 30]. The BSG model we employ here is based on the Connor-Stevens point neuron model [31]. The spike trains generated by the BSGs contain the odorant identity, odorant concentration, and concentration gradient information that the fly brain uses to make odorant valence decisions.

#### The odorant transduction process model

Two research groups have published widely different results on the OR transduction process in fruit flies [32–34]. As the exact signaling of the transduction cascade in fruit flies is still inconclusive, our approach focusses here on constructing a minimal transduction model. Called the fruit fly odorant transduction process (OTP) model, it extends the model proposed by [22, 23] by incorporating the essential features of temporal dynamics of other computational models, such as the one proposed by [21], while at the same time exhibiting the calcium dynamics of [26]. In the latter work, the temporal dynamics of fly’s OSN vanish in the absence of extracellular calcium. Notably, the calcium dynamics considered here constitutes a feedback mechanism that is similar to but also different from the one in the abstract model proposed by [28] (formally described by Eq (4)).

Olfactory transduction in fruit flies from airborne molecules to transduction current involves a number of steps [35, 36]: i) absorption of odorant molecules through the sensillum surface, binding between odorant molecules and odorant-binding proteins (OBPs), and diffusion of bound OBPs through the aqueous sensillar lymph to OSN dendrites, ii) odorant-receptor binding/dissociation, and iii) opening of ion channels that results in transduction current. The first step is known as the “peri-receptor” processing, the second step is referred as the bound receptor generator and the third step as the co-receptor channel. Taken together, they represent the fruit fly odorant transduction process.

We propose an olfactory transduction process model that consists of an *active receptor* model and a *co-receptor channel* model [29, 30]. The active receptor contains a peri-receptor model and a bound-receptor model. The peri-receptor process is modeled as a linear filter that describes the transformation of an odorant concentration waveform as odorant molecules diffuse through sensilla walls towards the OSN dendrites. The bound-receptor model encodes odorant identity and odorant concentration with a binding rate tensor, modulated by the odorant concentration profile, and a dissociation rate tensor. The odorant concentration profile is defined as the linearly weighted sum of the filtered odorant concentration and the filtered concentration gradient. Modulation is modeled here as a product. The co-receptor channel represents the ion channel gated by the atypical co-receptor (ORCO), Or83b. The calcium channel models the calcium dynamics, and provides a feedback mechanism to the co-receptor channel. The transduction current generator models the transmembrane current through the co-receptor channel.

#### The active receptor model

**Fig 2:**
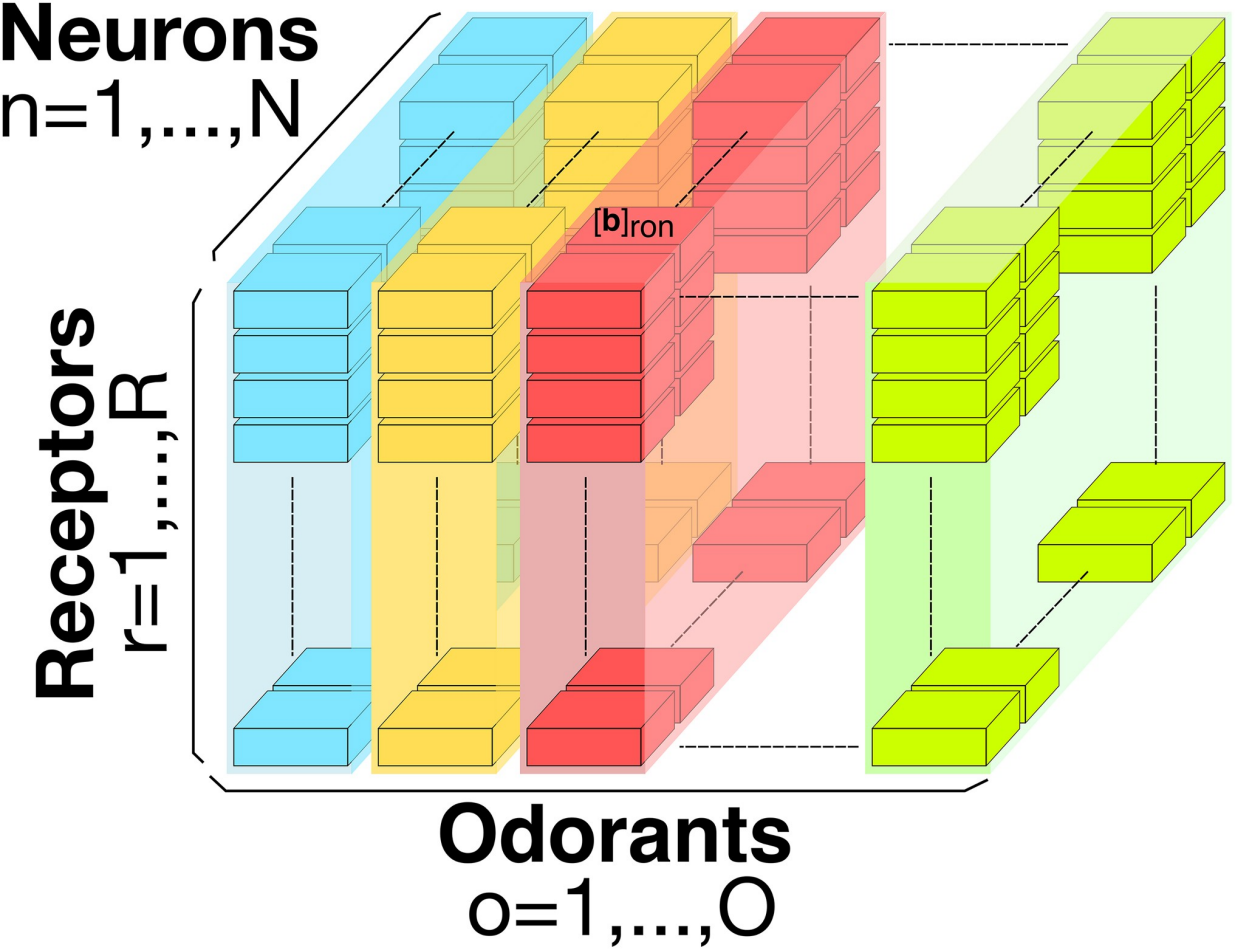
Three dimensional odorant-receptor binding rate tensor. For a given neuron *n* = 1, 2, …, *N*, the binding rate values are denoted by [**b**]_*ron*_, for all *r* = 1, 2, …, *R*, and *o* = 1, 2, …, *O*. For the fruit fly, the total number of neurons expressing the same receptor type is about *N* = 25, and the total number of receptor types is around *R* = 50. *O* is the number of all odorants that the fruit fly senses.

The fruit fly active receptor model quantifies the binding and the dissociation process between odorant molecules and odorant receptors. As introduced here, the model centers on the rate of change of the ratio of free receptors versus the total number of receptors [**x**_0_]_*ron*_ expressed by neuron *n*:

$$\begin{array}{c} \frac{d}{dt}{\left[x_{0}\right]}_{ron}=-{\left[b\right]}_{ron}\cdot {\left[v\right]}_{ron}\cdot {\left[x_{0}\right]}_{ron}+{\left[d\right]}_{ron}\cdot {\left[x_{1}\right]}_{ron}, \end{array}$$ (1)

where [**x**_1_]_*ron*_ is the ratio of ligand-bound receptors. Here *r* = 1, 2, …, *R*, is the receptor type, *o* = 1, 2…, *O*, denotes the odorant and *n* = 1, 2, …, *N*, denotes the neuron index. In Eq (1) above the ratios [**x**_0_]_*ron*_ and [**x**_1_]_*ron*_ are entries of the 3D tensors **x**_0_ and **x**_1_, respectively. The 3D tensor **b** with entries [**b**]_*ron*_ is called the *odorant-receptor binding rate* and models the association rate between an odorant and a receptor type. The 3D tensor **d** with entries [**d**]_*ron*_ denotes the odorant-receptor dissociation and models the detachment rate between an odorant and a receptor type. The 3D binding rate tensor **b** is graphically depicted in Fig 2 (A similar figure can be drawn for the dissociation rate tensor **d**). In what follows, the biding rate [**b**]_*ron*_ and the dissociation rate [**d**]_*ron*_, for a given odorant *o* and a given receptor type *r*, are assumed for simplicity to take the same value for all neurons *n* = 1, 2, …, *N*.

The tensor **v** with entry [**v**]_*ron*_ in Eq (1) is the odorant concentration profile and it is given by

$$\begin{array}{c} {\left[v\right]}_{ron}=\int_{R}h\left(t-s\right)u\left(s\right)ds+{\left[\gamma \right]}_{ron}\int_{R}h\left(t-s\right)du\left(s\right), \end{array}$$ (2)

if the right-hand-side is positive and zero otherwise. The right-hand-side is the weighted sum of the filtered odorant concentration *u* and the filtered concentration gradient *du*/*dt* with [**γ**]_*ron*_ denoting a weighting factor. We hypothesize that the impulse response of the linear filter *h* = *h*(*t*), t∈R (R denotes the set of real numbers), models the “peri-receptor” process that describes the transformation of odorant concentration waveform as odorant molecules diffuse through sensilla walls towards the dendrites of OSN [37]. For simplicity, the dependence of *h*(*t*) on the geometry of the sensillum and the diffusion of odorant molecules across the sensillar lymph is not considered here. *h*(*t*) in Eq (2) is the impulse response of a low-pass linear filter that is usually defined in the literature in frequency domain. Alternatively, *h*(*t*) is the solution to the second-order differential equation, see S1 Appendix. The output of the peri-receptor process (h*u)(*t*), i.e., the filtered concentration, then binds with the odorant receptors embedded into the OSN membrane via a nonlinear interaction approximated here by [**v**]_*ron*_.

The biophysical models of odorant transduction in the literature only consider the odorant concentration but not the (nonlinear) odorant concentration profile as the input to the transduction cascade [22, 23, 25, 26, 28]. The odorant concentration *profile* is critical for modeling 2D odorant encoding. As we will demonstrate in the *Methods and Materials* section, fitting the experimental data recorded in [16] using only the odorant concentration waveform leads to poor results.

We assume that receptors only have two states, either being “free” or “bound”, *i.e.,* [**x**_0_]_*ron*_+ [**x**_1_]_*ron*_ = 1. Then, Eq (1) amounts to,

$$\begin{array}{c} \frac{d}{dt}{\left[x_{1}\right]}_{ron}={\left[b\right]}_{ron}\cdot {\left[v\right]}_{ron}\cdot \left(1-{\left[x_{1}\right]}_{ron}\right)-{\left[d\right]}_{ron}\cdot {\left[x_{1}\right]}_{ron}. \end{array}$$ (3)

Eq (3) maps the input given by the product between the binding rate and the odorant concentration profile, and the dissociation rate, i.e., ([**b**]_*ron*_ ⋅ [**v**]_*ron*_, [**d**]_*ron*_), into the ratio of bound receptors [**x**_1_]. In what follows this map will be called the *bound-receptor generator*.

#### The co-receptor channel model

The fruit fly co-receptor channel and a calcium channel appear in a feedback configuration. Each of these components has its specific functionality. The *co-receptor channel* represents the ion channel gated by the atypical co-receptor (ORCO), Or83b. The *calcium channel* models the calcium dynamics, and provides a feedback mechanism to the co-receptor channel.

Next, we walk through each of the three equations of the co-receptor channel model. The key variables involved in the proposed odor transduction model are summarized in Table 1.

**Table 1:** Summary of the variables in the fruit fly odorant transduction model.

Variable	Description
*u*	odorant concentration waveform
*v*	odorant concentration profile
**b**	odorant-receptor binding rate
**d**	odorant-receptor dissociation rate
**x**_1_	ratio of ligand-bound receptors
**x**_2_	gating variable of the *co-receptor* channel
**x**_3_	gating variable of the calcium channel
**I**	transduction current generated by the *co-receptor* channel

1. The rate of change of the *gating* variable of the co-receptor channel [**x**_2_]_*ron*_:
  $$\begin{array}{c} \frac{d}{dt}{\left[x_{2}\right]}_{ron}=\alpha_{2}\cdot {\left[x_{1}\right]}_{ron}\left(1-{\left[x_{2}\right]}_{ron}\right)-\beta_{2}\cdot {\left[x_{2}\right]}_{ron}-\kappa \cdot {\left[x_{2}\right]}_{ron}^{2/3}\cdot {\left[x_{3}\right]}_{ron}^{2/3}, \end{array}$$ (4)
  where *α*_2_ and *β*_2_ are scalars indicating the rate of activation and deactivation of the gating variable, respectively, and $\kappa \cdot {\left[x_{2}\right]}_{ron}^{2/3}\cdot {\left[x_{3}\right]}_{ron}^{2/3}$ models the calcium feedback with *κ* a constant. The feedback coupling between [**x**_2_]_*ron*_ and [**x**_3_]_*ron*_ that arises in the co-receptor channel model considered here is similar to the one proposed by De Palo et al. [28]. However, it also differs from it in two important ways. First, the input to the co-receptor channel is the ratio of the ligand-bound receptors [**x**_1_]_*ron*_ driven, among others, by the odorant concentration profile [**v**]_*ron*_, while De Palo et al. used the odorant concentration *u*. Second, the feedback mechanism is based on the fractional power 2/3 for the variables [**x**_2_]_*ron*_ and [**x**_3_]_*ron*_, while De Palo et al. used the variables raised to power 1 in their feedback model. The fractional power is key in facilitating the encoding of the filtered concentration gradient.
2. The rate of change of the *gating* variable of the calcium channel [**x**_3_]_*ron*_:
  $$\begin{array}{c} \frac{d}{dt}{\left[x_{3}\right]}_{ron}=\alpha_{3}\cdot {\left[x_{2}\right]}_{ron}-\beta_{3}\cdot {\left[x_{3}\right]}_{ron}, \end{array}$$ (5)
  where *α*_3_ and *β*_3_ are scalars indicating the rate of increase and decrease of the gating variable.
3. Finally, the transduction current [**I**]_*ron*_ is given by:
  $$\begin{array}{c} {\left[I\right]}_{ron}=\frac{{\left[x_{2}\right]}_{ron}^{p}}{{\left[x_{2}\right]}_{ron}^{p}+c^{p}}\cdot I_{max}, \end{array}$$ (6)
  where *p* and *c* are scalars, and *I*_*max*_ denotes the maximal amplitude of the current through the *co-receptor* channel, whose value is empirically determined through parameter sweeping.

Combining the equations introduced above, we rewrite the odorant transduction process model in compact form as

$$\begin{array}{rr} {\left[v\right]}_{ron} & =Re\left(\int_{\mathbb{R}}h(t-s)u(s)ds+{\left[\gamma \right]}_{ron}\int_{\mathbb{R}}h(t-s)du(s)\right)\\ {\left[\begin{array}{c} {\dot{x}}_{1}\\ {\dot{x}}_{2}\\ {\dot{x}}_{3} \end{array}\right]}_{ron} & =\left(\begin{array}{c} {\left[b\right]}_{ron}\cdot {\left[v\right]}_{ron}\cdot (1-{\left[x_{1}\right]}_{ron})-{\left[d\right]}_{ron}\cdot {\left[x_{1}\right]}_{ron}\\ \alpha_{2}\cdot {\left[x_{1}\right]}_{ron}(1-{\left[x_{2}\right]}_{ron})-\beta_{2}\cdot {\left[x_{2}\right]}_{ron}-\kappa \cdot {\left[x_{2}\right]}_{ron}^{2/3}\cdot {\left[x_{3}\right]}_{ron}^{2/3}\\ \alpha_{3}\cdot {\left[x_{2}\right]}_{ron}-\beta_{3}\cdot {\left[x_{3}\right]}_{ron} \end{array}\right)\\ {\left[I\right]}_{ron} & =\frac{{\left[x_{2}\right]}_{ron}^{p}}{{\left[x_{2}\right]}_{ron}^{p}+c^{p}}\cdot I_{max}, \end{array}$$ (7)

for all *r* = 1, 2, …, *R*, *o* = 1, 2, …, *O* and *n* = 1, 2, …, *N*. *Re* above denotes the rectification function.

#### Biophysical spike generator model

We restricted our choice of OSN spiking mechanism to biophysical spike generators (BSG) such as the Hodgkin-Huxley, the Morris-Lecar, and the Connor-Stevens point neuron models. The firing rate of biological OSNs spans continuously between 0 to 300 spikes per second. The Hodgkin-Huxley model displays a “type II” excitability [38] with a discontinuous frequency-current curve. The Morris-Lecar model has “type I” excitability that leads to a continuous frequency-current curve, but its maximum spike rate is below 50 spikes per second [38]. The Connor-Stevens model is our model of choice. It exhibits a “type I” excitability and a firing rate of up to 300 spikes per second. In S1 Appendix we only discuss the Connor-Stevens (CS) point neuron model.

### The odorant space

As already discussed, the odorant identity is modeled by the binding rate tensor **b** and the dissociation rate tensor **d**, while the odorant concentration waveform is denoted by *u* = *u*(*t*), *t* ≥ 0. Thus the abstract odorants (elements) of the odorant space are the tuples (**b**, **d**, *u*). Each OTP (indexed here as (*r*, *o*, *n*)) represents an abstract odorant as the pair ([**b**]_*ron*_[**v**]_*ron*_, [**d**]_*ron*_), where **v** = **v**(*t*), *t* ≥ 0, denotes the concentration profile.

The three entities above, the concentration profile, the binding rate tensor, and the dissociation rate tensor, jointly determine the level of complexity of the odorant space, and thereby, the level of complexity of the spatio-temporal encoding circuits.

### Biological validation of the OSN model

The essential functionality of OSNs is to jointly encode both odorant identity and odorant concentration. To address these two functional characteristics we modeled each OSN as an OTP/BSG cascade. To validate our approach, we examine here the response of the OSN model to odorant waveforms that were previously used in experiments with different odorants and receptors, and compare the model responses with electrophysiological recordings.

We first optimized all the parameters of the OTP model, including the binding rate and dissociation rate, for the (*acetone*, *Or59b*) pair using electrophysiological recordings of two datasets obtained in response to pulse-like odorant waveforms and a white noise odorant waveform (see Methods and materials section).

Second, we evaluate the temporal response of the *Or59b* OSN model to acetone with a multitude of stimuli, including step, ramp and parabola waveforms. We further evaluate the model with staircase and white noise waveforms. Our results show that the model closely matches the complex temporal response of *Or59b* OSN electrophysiological recordings. Lastly, we evaluate the affinity and dissociation rate for different odorant-receptor pairs including (*methyl butyrate*, *Or59b*) and (*butyraldehyde*, *Or7a*).

#### Estimating the binding and dissociation rates for (*acetone*, *Or59b*)

As already mentioned above, we first fitted the value of every parameter in the OTP model for (*acetone*, *Or59b*) in two different settings. In the first setting, we ran an optimization procedure (see Methods and materials section) with a white noise odorant waveform, a set of pulse-like odorant waveforms (see Dataset 1 in S1 Appendix), and the corresponding OSN spike train recordings. In the second setting, we used the same white noise odorant waveform and a different set of pulse-like odorant waveforms (see Dataset 2 in S1 Appendix). In both settings, the optimization procedure of the OTP/BSG cascade uses the recordings in response to the white noise odorant waveform for fitting the transient response and the recordings of the response to pulse-like waveforms for fitting the steady state response.

**Fig 3:**
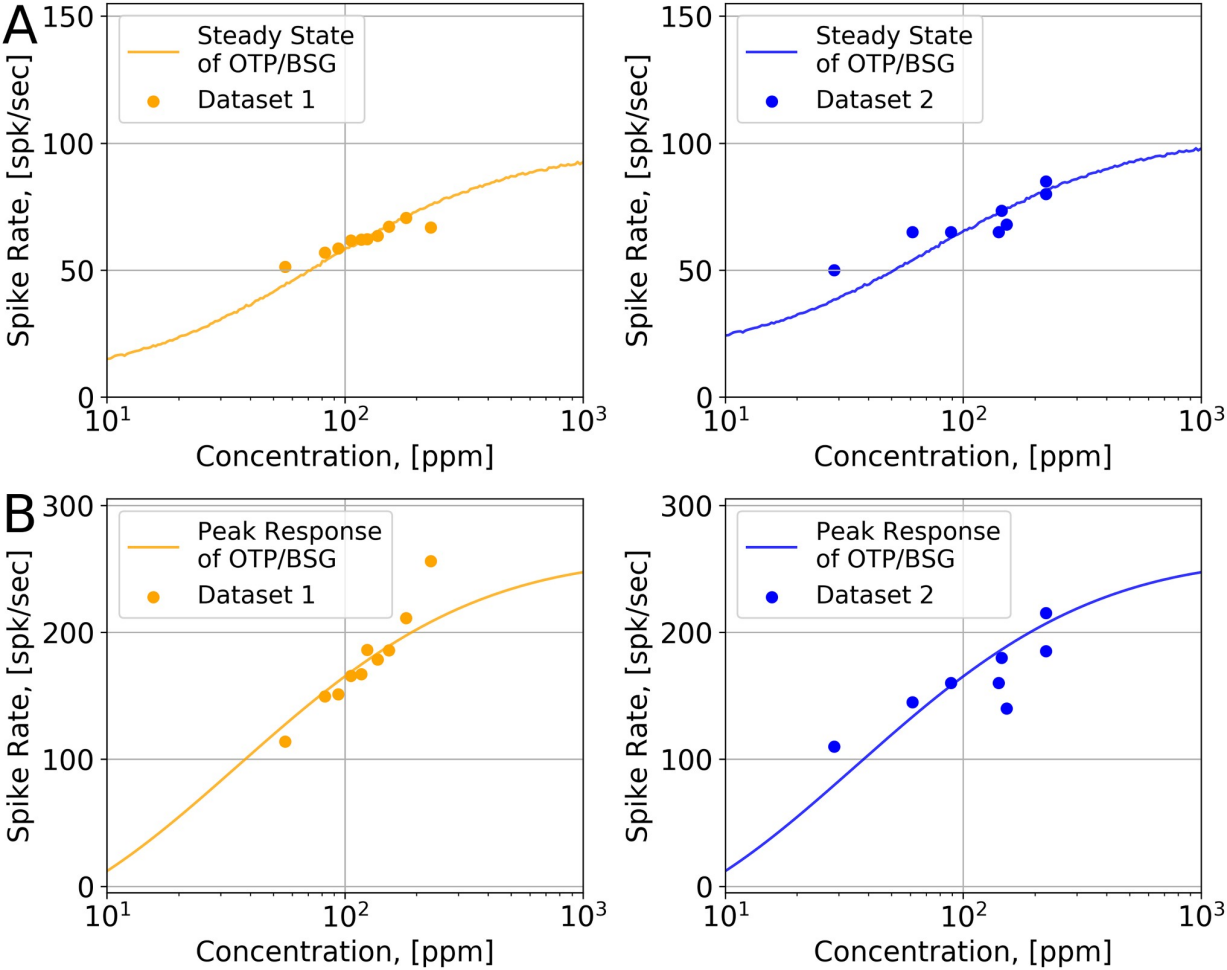
Estimation of the binding rate and the dissociation rate for (*Or59b*,*acetone*). (A) Estimation of the binding value for two datasets. The source of the two datasets is given in S1 Appendix. Both datasets contain the recordings of OSNs expressing *Or59b* in response to acetone step waveforms. (Left) Dataset 1: Estimated binding rate is 2.17 ⋅ 10^−2^ ⋅ (*ppm* ⋅ *s*)^−1^; (Right) Dataset 2: Estimated binding rate is 2.11 ⋅ 10^−2^ ⋅ (*ppm* ⋅ *s*)^−1^; (B) Estimation of the dissociation rate for two datasets. (Left) Dataset 1: Estimated dissociation rate is 2.94 ⋅ *s*^−1^; (Right) Dataset 2: Estimated dissociation rate is 2.65 ⋅ *s*^−1^.

We estimated binding and dissociation rates for two datasets. For Dataset 1, the estimated biding and dissociation rates are 2.17 ⋅ 10^−2^ ⋅ (*ppm* ⋅ *s*)^−1^ and 2.94 ⋅ *s*^−1^, respectively, and for Dataset 2, the estimated biding and dissociation rates are 2.11 ⋅ 10^−2^ ⋅ (*ppm* ⋅ *s*)^−1^ and 2.65 ⋅ *s*^−1^, respectively. The steady state responses of the OTP/BSG model and the OSN recordings are shown in Fig 3A, and the peak responses of the OTP/BSG model and the OSN recordings are shown in Fig 3B.

#### Evaluating the temporal response of the *Or59b* OSN model to *acetone*

To evaluate temporal response, we stimulated the OSN model with multiple odorant stimuli that were previously used in experiments designed for characterizing the response to *acetone* of OSNs expressing *Or59b*. For all OTP models considered below we set the odorant-receptor binding rate to 2.17 ⋅ 10^−2^ ⋅ (*ppm* ⋅ *s*)^−1^ and the odorant-receptor dissociation rate to 2.94 ⋅ *s*^−1^.

#### Response of the *Or59b* OSN model to step, ramp and parabola *acetone* waveforms

**Fig 4:**
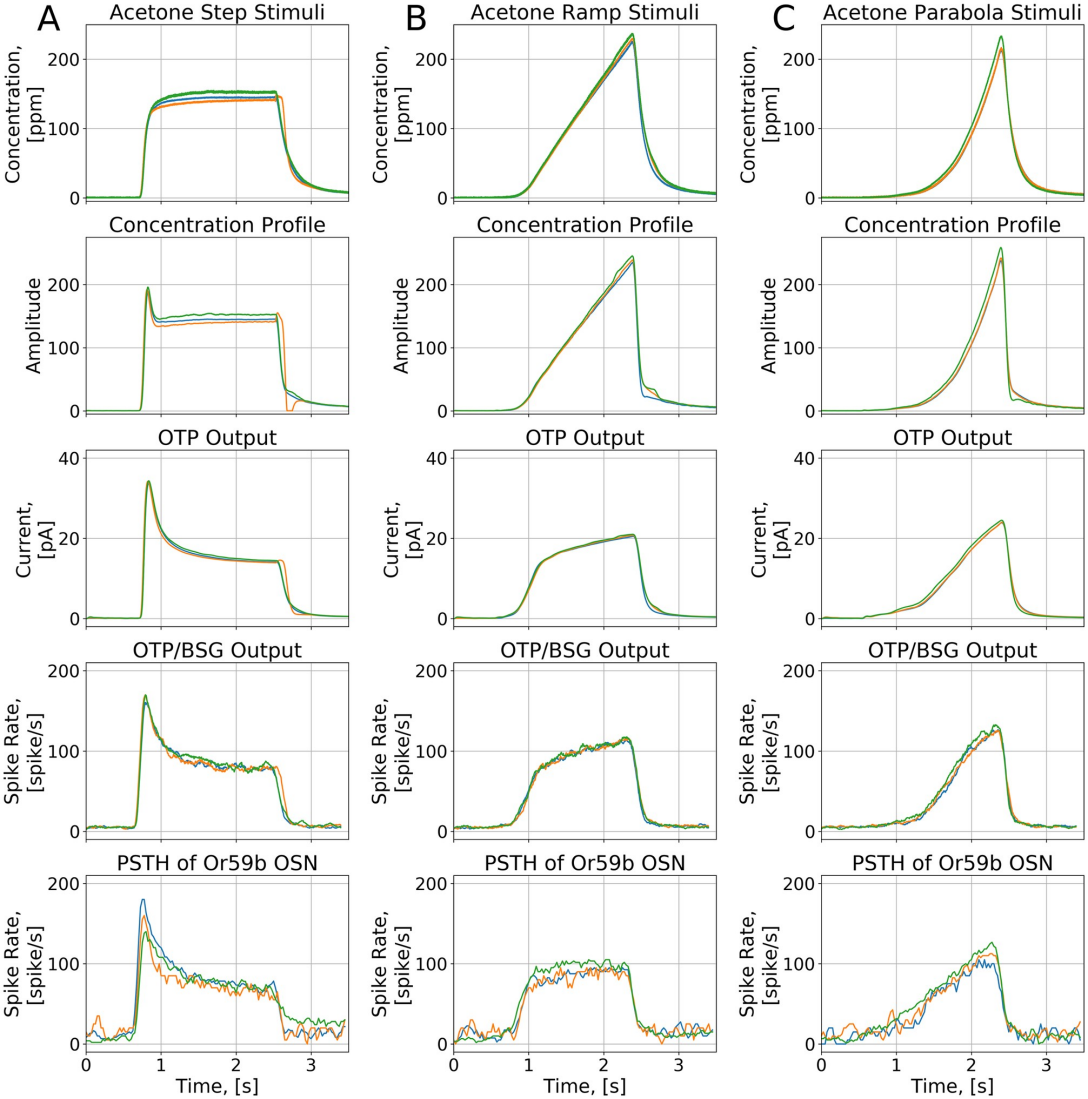
Characterization of the OTP/BSG cascade in response to step, ramp, and parabola stimuli. Odorant: *acetone*, receptor: *Or59b*. The stimulus waveforms are identical to the ones used in [17]. The odorant-receptor binding and dissociation rates were set to 2.17 ⋅ 10^−2^ ⋅ (*ppm* ⋅ *s*)^−1^ and 2.94 ⋅ *s*^−1^, respectively. (A) Step stimuli. (B) Ramp stimuli. (C) Parabola stimuli. (First row) Stimulus waveforms. (Second row) The odorant concentration profile. (Third row) The transduction current at the output of the OTP model. (Forth row) PSTH computed from the output of the OTP/BSG output. (Fifth row) PSTH of the spike train generated by the *Or59b* OSN in response to the stimulus waveforms (using the original raw data published in [17]).

We first evaluated the response of the *Or59b* OSN model to step, ramp, and parabola stimulus waveforms as shown in the first row of Fig 4. The temporal response of the OTP/BSG cascade (the fourth row of Fig 4) is similar to the one of the OTP model (the third row of Fig 4). For step stimuli, the OTP/BSG cascade generates a chair-shaped response by first picking up the gradient of the concentration right after the onset of the odorant, and then gradually dropping down to a constant value, that encodes the step value of the amplitude. For ramp stimuli, the initial response of the OTP/BSG cascade rapidly increases, and then it plateaus as the gradient of ramp stimuli becomes constant. Lastly, for the parabola stimuli, the response of the OTP/BSG cascades resembles a ramp function, that corresponds to the gradient of parabola stimulus waveforms.

Furthermore, we also compared the PSTH of the spike trains generated by the OTP/BSG cascade with the PSTH of an *Or59b* OSN obtained from electrophysiology recordings in response to acetone concentration waveforms [17]. As shown in Fig 4, the OTP/BSG closely matches the odorant response of the *Or59b* OSN with the mean squared error between the model output and OSN recording equal to 13.84 for the step stimuli, 14.92 for the ramp stimuli, and 13.71 for the parabola stimuli (see also the Methods and materials section).

#### Response of the *Or59b* OSN model to white noise *acetone* waveforms

To further compare the response of the OTP/BSG cascade with the *Or59b* OSN response, we stimulated OTP/BSG cascades with white noise stimuli, and compared the PSTH of the model with the one from experimental recordings. The white noise stimulus was previous used in the experimental setting of [16] for characterizing the response of *Or59b* OSNs to acetone.

**Fig 5:**
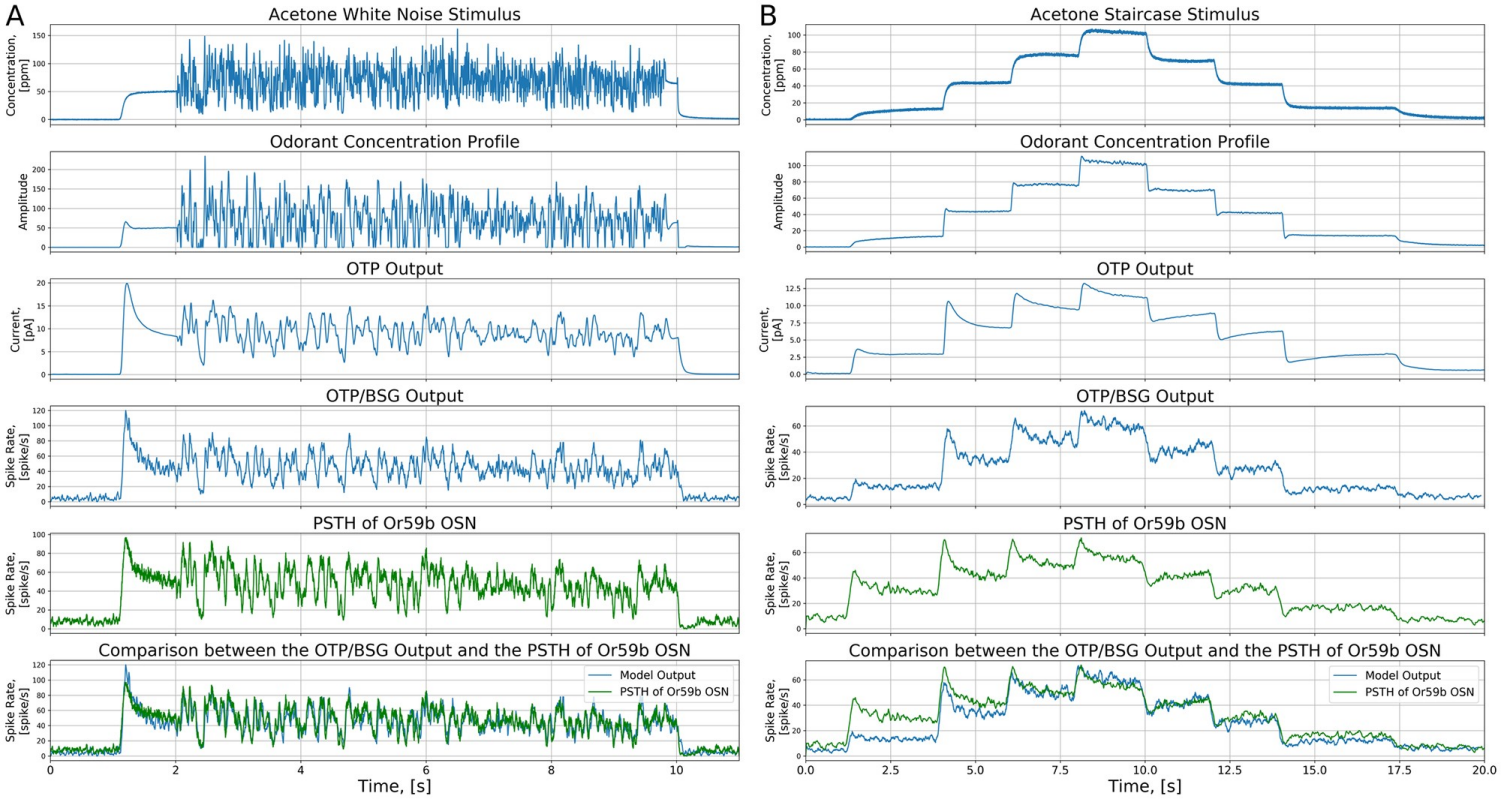
Characterization of the OTP/BSG cascade in response to white noise and staircase stimuli. The two stimuli were previously used in [16]. Odorant: *acetone*, receptor: *Or59b*. The odorant-receptor binding and dissociation rates were set to 2.17 ⋅ 10^−2^ ⋅ (*ppm* ⋅ *s*)^−1^ and 2.94 ⋅ *s*^−1^, respectively. (A) White noise. (B) Staircase. (First row) White noise and staircase stimuli. (Second row) The odorant concentration profile. (Third row) The output of the OTP model. (Forth row) The PSTH of the spike train generated by the OTP/BSG cascade. (Fifth row) The PSTH of the spike train of the recorded OSN (using the original raw data published in [16]). (Sixth row) Comparison between the PSTHs at the output of the OTP/ BSG cascade and the recorded OSN.

The output of each of the stages of the *Or59b* OSN model are shown in Fig 5A. The odorant onset at around 1 second is picked up by the odorant concentration profile (see the the second row of Fig 5A. In addition, the white noise waveform between 2 and 10 second is smoothed out. The smoothing effect is due to the peri-receptor process filter. The OTP model further emphasizes the gradient encoding (the third row of Fig 5A), and predominantly defines the temporal response of the OSN model to white noise stimuli. The BSG output follows the OTP output, as the BSG is simply a sampling device. Lastly, we compare the model output and the PSTH from OSN recordings in [16] (the fifth and the sixth rows of Fig 5A). The *Or59b* OSN model output PSTH closely matches the PSTH obtained from recordings with the mean squared error between the model output and OSN recording equal to 8.94 (see also the Methods and materials section).

The peri-receptor process filter is critical in processing the white noise waveforms, but less critical in processing the static waveforms discussed in this work. The filter prevents the model from overemphasizing the gradient of the white noise waveforms. In absence of this filter, the response of the OTP/BSG cascade is severely limited in matching the response of *Or59b* OSN [16] to acetone waveforms.

#### Response of the *Or59b* OSN model to staircase *acetone* waveforms

Next, we stimulated OTP/BSG cascade with the staircase waveform that was previously used in experiments [16], evaluated the PSTH from the resultant spike sequences, and compared the model PSTH to the one from experimental recordings.

As shown in the second row of Fig 5B, the filter *h*(*t*) in Eq (2) has negligible effect on the odorant concentration profile since the staircase is smooth unlike the white noise stimulus discussed above. The encoding at jump times is strongly sharpened by the OTP. Overall, the fruit fly OTP/BSG cascade indeed encodes both the concentration and concentration gradient. In particular, at each upward concentration jump, the PSTH of the OSN launches an upward overshoot and then drops down to a saturation point. In addition, at each downward concentration jump, the same PSTH drops down first to a downward jump and then bounces back.

The gradient term in Eq (2) introduces a small upward overshoot in the concentration profile at each concentration jump as shown in the second row of Fig 5B. However, the amplitude of these overshoots is small compared to the overshoots at PSTH jump times. We note here that, the OTP model further amplifies the overshoot in the concentration profile to a larger value of the transduction current, as shown in the third row of Fig 5B. The latter effect leads to the significant overshoot of the PSTH. In short, the OSN model closely reproduces the temporal response of *Or59b* OSNs for all tested stimuli. This suggests that the OPT/BSG cascade has the desired level of complexity to effectively model the fruit fly OSNs in response to the mono-molecular odorants.

#### Evaluating affinity, binding and dissociation rates of other (*odorant*, *receptor*) pairs

We next interrogate the role of the binding and dissociation rates in the OTP/BSG cascade. For a given receptor type and two odorants with different binding rates and the same dissociation rate, responses of the OTP/BSG cascade are identical if the waveforms of two odorants only differ by a scaling factor that is the reciprocal of the ratio of the biding rates. This follows from Eq (3).

#### Response of *Or59b* OSNs to two different odorants

**Fig 6:**
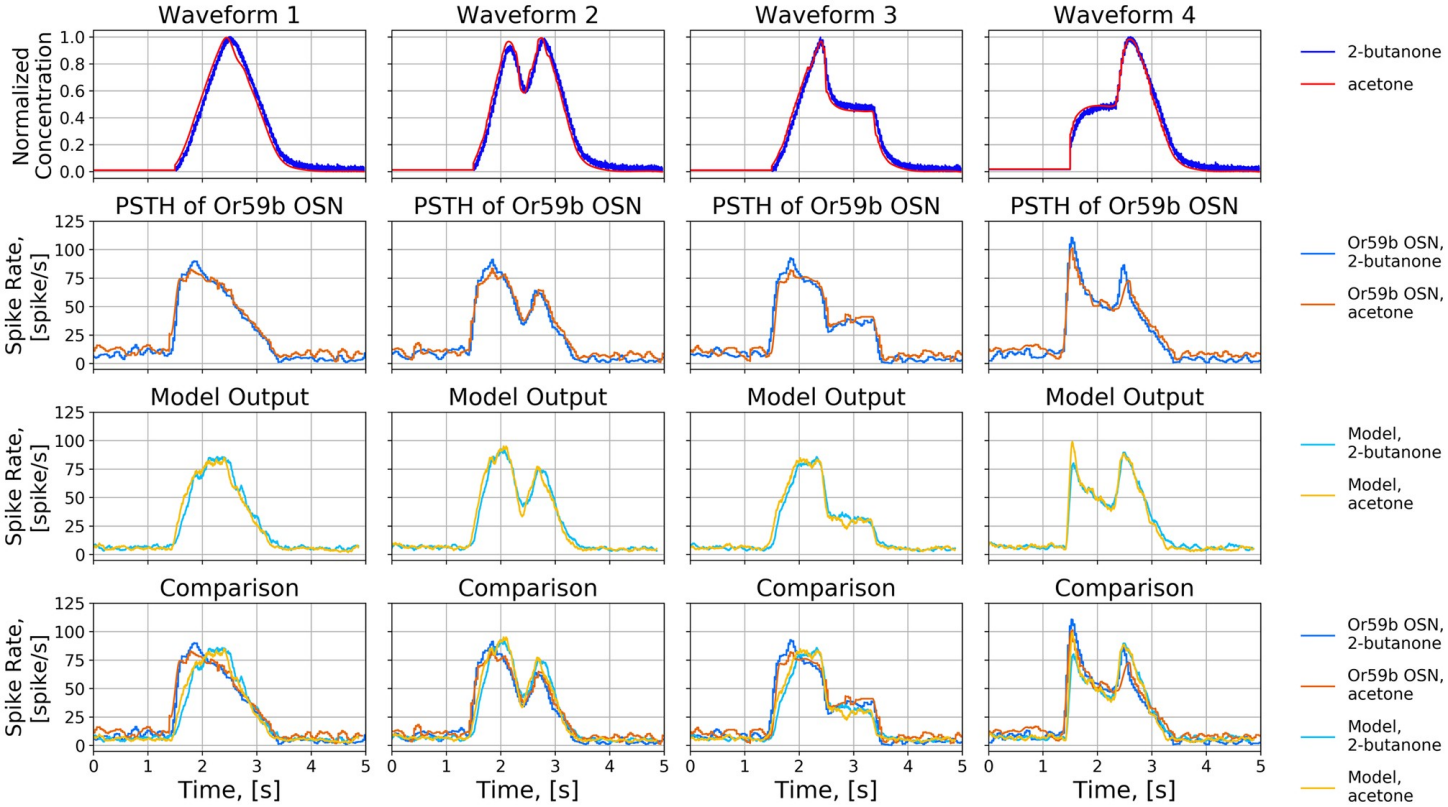
Comparison of the responses of an Or59b OSN to four concentration waveforms of *acetone* and *2-butanone*. (First row) Four normalized concentration waveforms. Each normalized waveform is scaled by 100 for *acetone* and by 10 for *2-butanone*. (Second row) PSTH of the *Or59b* OSN in response to the two odorants (using the original raw data published [39]). (Third row) The PSTH of the spike train generated by the OTP/BSG cascade. (Forth row) Comparison between the PSTHs at the output of the OTP/BSG cascade and the recorded OSN in [39].

We verify the prediction mentioned above by stimulating the *Or59b* OSN model with two odorant stimuli, *acetone* and *2-butanone*, paired with four concentration waveforms, and compare the responses with the experimental recordings in [39]. As shown in the first row of Fig 6, the two odorants have identical normalized waveforms scaled by two different factors, 100 and 10. The binding rate of *acetone* and *2-butanone* were estimated to be 2.17 ⋅ 10^−2^ ⋅ (*ppm* ⋅ *s*)^−1^ and 2.18 ⋅ 10^−1^ ⋅ (*ppm* ⋅ *s*)^−1^, respectively, and the dissociation rate of the two odorants were estimated to be 2.94 ⋅ *s*^−1^ and 2.94 ⋅ *s*^−1^, respectively.

The two odorant stimuli elicit almost exactly the same response from the *Or59b* OSN recordings (see the second row of Fig 6) as well as from the output of the OTP/BSG cascade (see the third row of Fig 6). The difference in binding rate for *acetone* and *2-butanone* is perfectly counterbalanced by the scaling factors of the odorant waveforms in Fig 6. In addition, the output of the OTP/BSG cascade closely reproduces the PSTH of the *Or59b* OSN as shown in the forth row of Fig 6 (see the Methods and materials section for a quantitative comparison between the model output and the OSN recordings).

#### Evaluating the odorant-receptor response of the OTP/BSG cascade

We further investigated the role of the binding rate using three odorant-receptor pairs that were previously used in experimental settings [39]. In addition to the binding rate estimated for (*acetone*, *Or59b*), we applied the estimation algorithm in Box to two additional odorant-receptor pairs using the original raw data presented in [39]:

1. for (*methyl butyrate*, *Or59b*) an affinity value 4.264 ⋅ 10^−3^ ⋅ (*ppm*)^−1^ and a dissociation value 3.788 ⋅ *s*^−1^ were obtained from the steady-state spike rate at 87 spikes per second and the peak spike rate at 197 spikes per second in response to a constant stimulus with amplitude 20 ppm;
2. for (*butyraldehyde*, *Or7a*) an affinity value of 7.649 ⋅ 10^−4^ ⋅ (*ppm*)^−1^ and a dissociation value 8.609 ⋅ *s*^−1^ were obtained from the steady-state spike rate at 43 spikes per second and the peak spike rate at 101 spikes per second in response to a constant stimulus with amplitude 173 ppm;

We simulated the OSN model for each of the three odorant-receptor pairs with three types of stimuli, step, ramp, and parabola. The binding and dissociation rates for different odorant-receptor pairs above were separately set.

**Fig 7:**
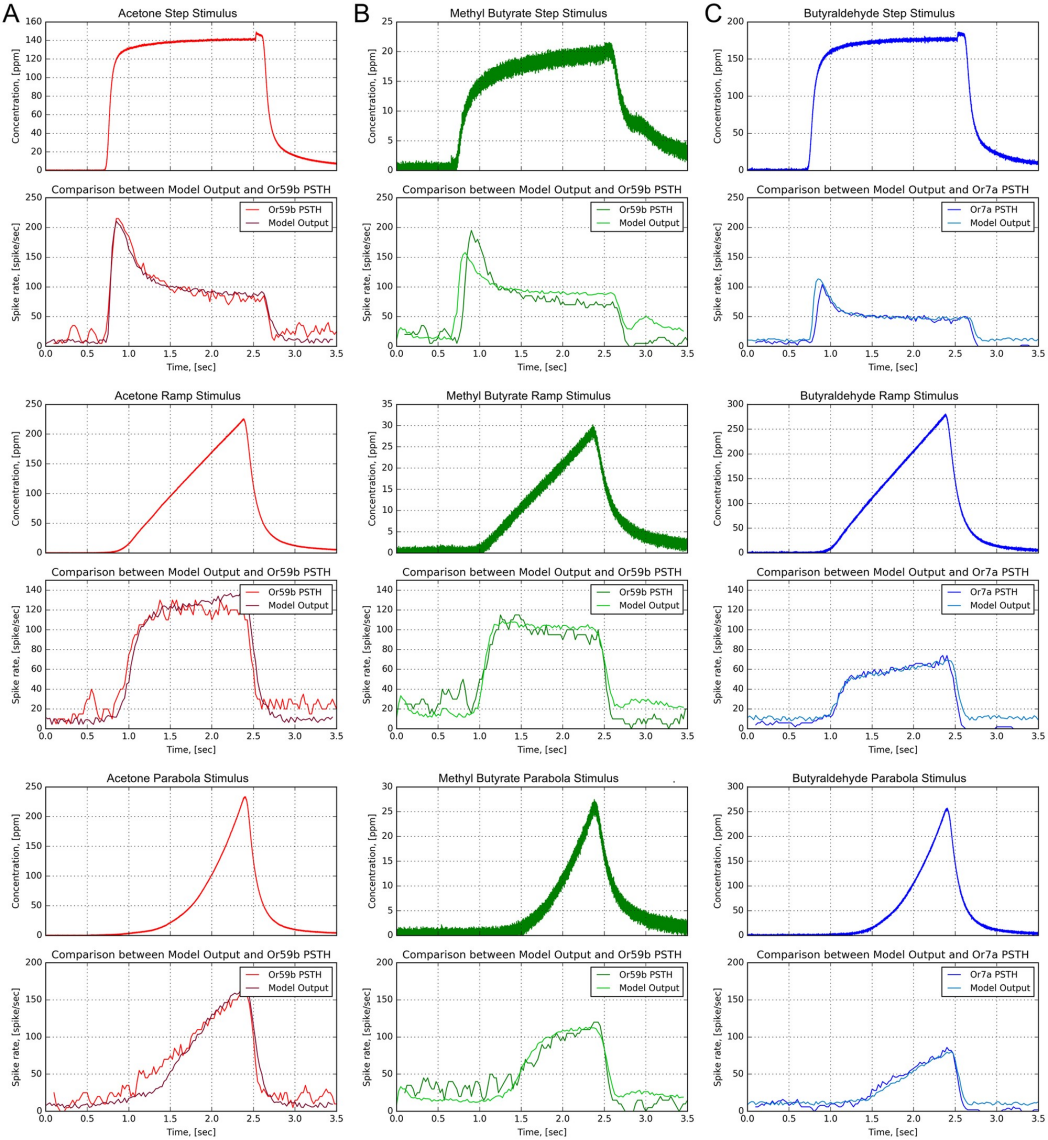
Characterization of the OTP/BSG cascade with multiple odorants and receptor types. Three odorant-receptor pairs are tested: 1) *Or59b* and *acetone*, 2) *Or59b* and *methyl butyrate*, and 3) *Or7a* OSN and *butyraldehyde*. (A) *Or59b* OSN in response to *acetone*. (B) *Or59b* OSN in response to *methyl butyrate*. (C) *Or7a* OSN in response to *butyraldehyde*. (Odd rows) Odorant stimuli. (Even rows) PSTH from the model output and experimental recordings [39] (using the original raw data published in [39]).

As shown in Fig 7, with only the change in the value of the binding and dissociation rates, the OSN model closely matches the OSN’s response for all three tested odorant-receptor pairs. The results in Fig 7 suggests that a pair of binding and dissociation rates is capable of closely matching the temporal response of different temporal odorant concentration waveforms.

### Estimating the *odorant*-*receptor* affinity matrix with DoOR datasets

**Fig 8:**
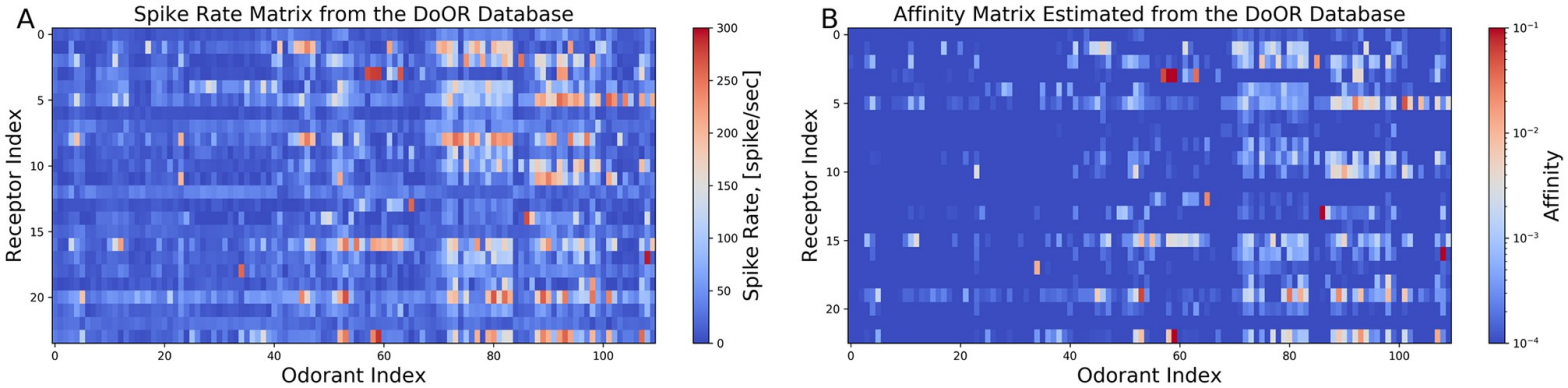
Estimating the odorant-receptor affinity matrix. (A) Spike rate matrix from the DoOR database containing 24 odorant receptors and 110 odorants. The data was originally published in [2]. Each column represents an odorant, and each row represents an OSN receptor type. (B) Each entry of the affinity matrix is estimated from each entry of the spike rate matrix using the inverse of the function empirically determined with the estimation algorithm in Box 1. Note the log-scale color map for the affinity values.

The DoOR database integrates OSN recordings obtained with different measurement techniques [4, 5], including *in situ* spike counts [3, 15, 40] and calcium activity [41], among others. *Spike counts* are directly available from OSN spike train recordings. Relating calcium activity to spike activity is, however, error prone. We consequently focus here on the odorant-OSN response datasets of the DoOR database that contain spike count information [2]. These datasets currently contain spike counts of 24 OSN groups in response to 110 odorants with a constant amplitude of 100 pm. The spike count is color coded and depicted Fig 8A. Unlike the aforementioned OSN recordings for which the temporal response is available for fitting all parameters in the OTP model, the DoOR database provides a single data point for every odorant-receptor pair, and hence the degree of freedom for fitting is limited. Due to this limitation, we estimate only the affinity and fix, for every odorant-receptor pair in the DoOR database, the value of the other parameters of the OTP model as listed in the *Methods and Materials* section. By employing the estimation algorithm in Box 1, we empirically estimated the affinity value for all 110 ⋅ 24 = 2, 640 odorant-receptors pairs. The estimated affinity corresponding to each entry of the spike rate matrix shown in Fig 8A is depicted in Fig 8B.

In summary, the binding and dissociation rate model together with the rest of the OTP/BSG cascade define a family of OSN models, and provide the scaffolding for studying the neural coding for odorant *identity* and odorant *concentration* in temporal domain at the OSN antennae population level.

### Evaluating the temporal response of the AMP LPU

We investigated the temporal response of the OTP/BSG cascade to various odorant waveforms, including step, ramp, parabola, staircase, and white noise waveforms. In addition, we biologically validated the cascade with electrophysiological recordings of OSNs by demonstrating that the cascade is capable of reproducing the complex temporal responses of OSNs for multiple odorant receptor pairs.

Here, we study the temporal response of an AMP LPU, that consists of 50 OSN groups [42, 43] on either side of the fruit fly brain. Each of the 50 OSN groups consists of 25 OTP/BSG cascades (neurons) that express an unique receptor type. We tested the AMP LPU with the same staircase waveform as in Fig 5B. For an assumed odorant, we assigned the same odorant-receptor affinity to OTPs in the same OSN group. The value of the affinity for each of the 25 OTP ranges between 2 ⋅ 10^−4^ ⋅ (*ppm*)^−1^ and 10^−2^ ⋅ (*ppm*)^−1^ with a step size of 2 ⋅ 10^−4^ ⋅ (*ppm*)^−1^. The dissociation rate for all OTP models was set to 10^2^. For simplicity, we used the same set of parameters for all cascades across all OSN groups. The parameters of the OTP model are given in the *Methods and Materials* section, and the parameters of the BSG model are listed in S1 Appendix. From the spike sequences generated by the 25 cascades we evaluated the PSTH for each of the 50 OSN groups.

**Fig 9:**
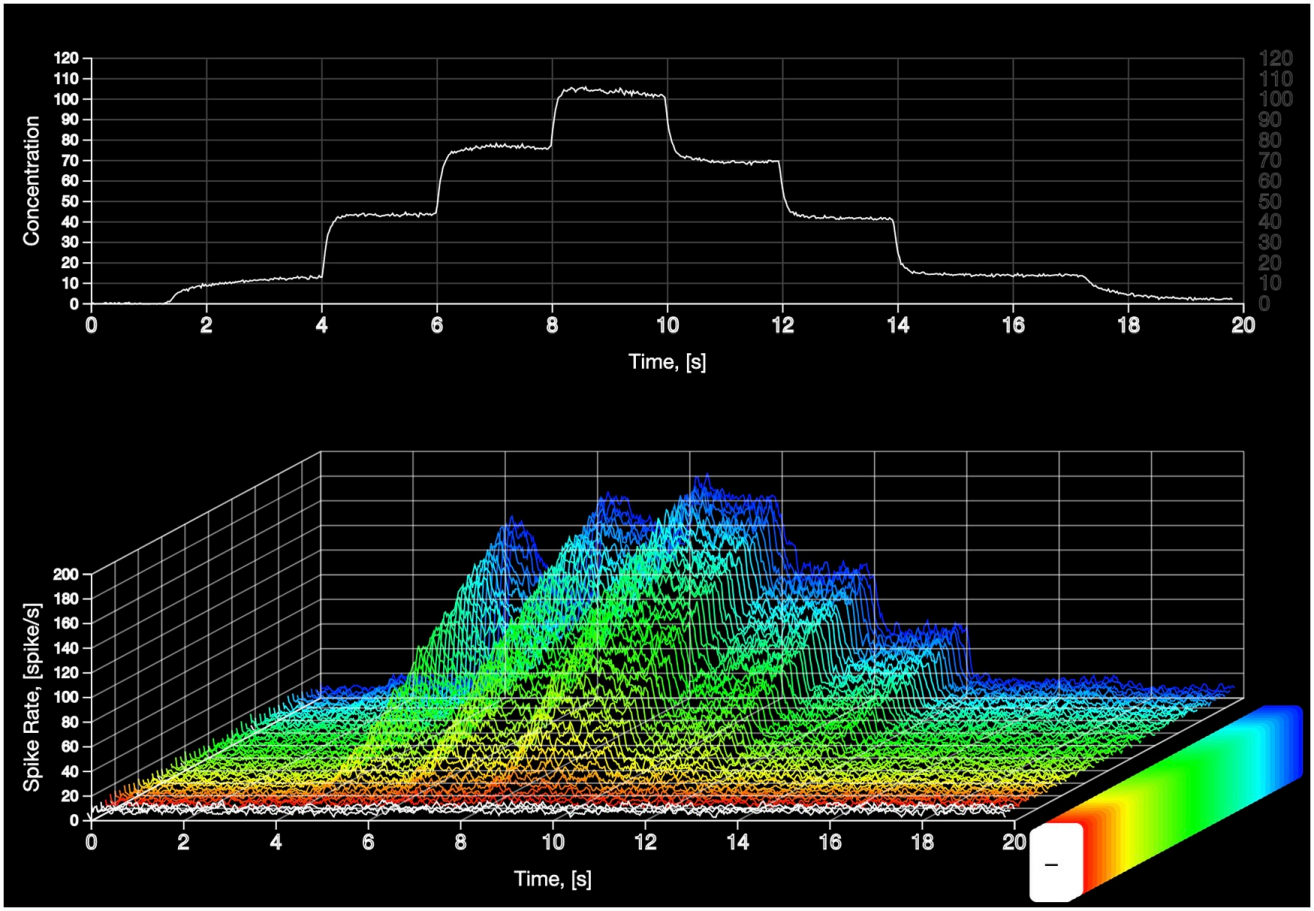
Preview of the animation demonstrating the AMP LPU in response to a staircase concentration waveform. The animation in S1 Video was rendered by NeuroGFX [44]. Each of the OSN groups consists of 25 fruit fly OTP/BSG cascades. The PSTH for each of the OSN groups was evaluated from the spike sequences generated by 25 cascades. The affinity for each of the 50 OSN groups was assumed to be ranging between 2 ⋅ 10^−4^ ⋅ (*ppm*)^−1^ and 10^−2^ ⋅ (*ppm*)^−1^ with a step size 2 ⋅ 10^−4^ ⋅ (*ppm*)^−1^. The dissociation rate for all OTP models was set to 10^2^. The rest of parameters of both OTP and BSG are given in the *Methods and Materials* section and S1 Appendix, respectively. (top) Staircase odorant stimulus. (bottom) 3D view of 50 OTP/BSG PSTHs. The response curves are sorted in ascending order according to the amplitude of the binding rate.

We visualize the 50 PSTHs and provide the preview to the animation in Fig 9. The animation is rendered by NeuroGFX, a key component of FFBO [44] (both NeuroGFX and FFBO are briefly presented in the *Methods and Materials* section). As shown, the top plot in the animation (and in Fig 9) shows the staircase odorant waveform, and the bottom plot shows the 3D view of the 50 PSTHs.

The resultant PSTHs exhibit distinct temporal responses across different OSN groups. Both the concentration and concentration gradient of odorants with (overall) high binding rate values is 2D encoded.

For receptors with extremely low (overall) binding rate values, the OTP/BSG cascade generates an output only after the concentration amplitude exceeds a certain value. For example, as shown in Fig 9, OSNs expressing receptors marked with orange color (See also Fig. A3 in S1 Appendix for a 2D preview with the orange color highlighted.) remain silent in the time interval between 2 to 6 seconds under a weak amplitude concentration stimulation. They start reacting to the odorant stimulus after 8 seconds as the amplitude increases from 80 ppm to 100 ppm. This phenomenon is consistent with the finding in [2] that a receptor only reacts to an odorant when the concentration amplitude exceeds a certain threshold value.

**Fig 10:**
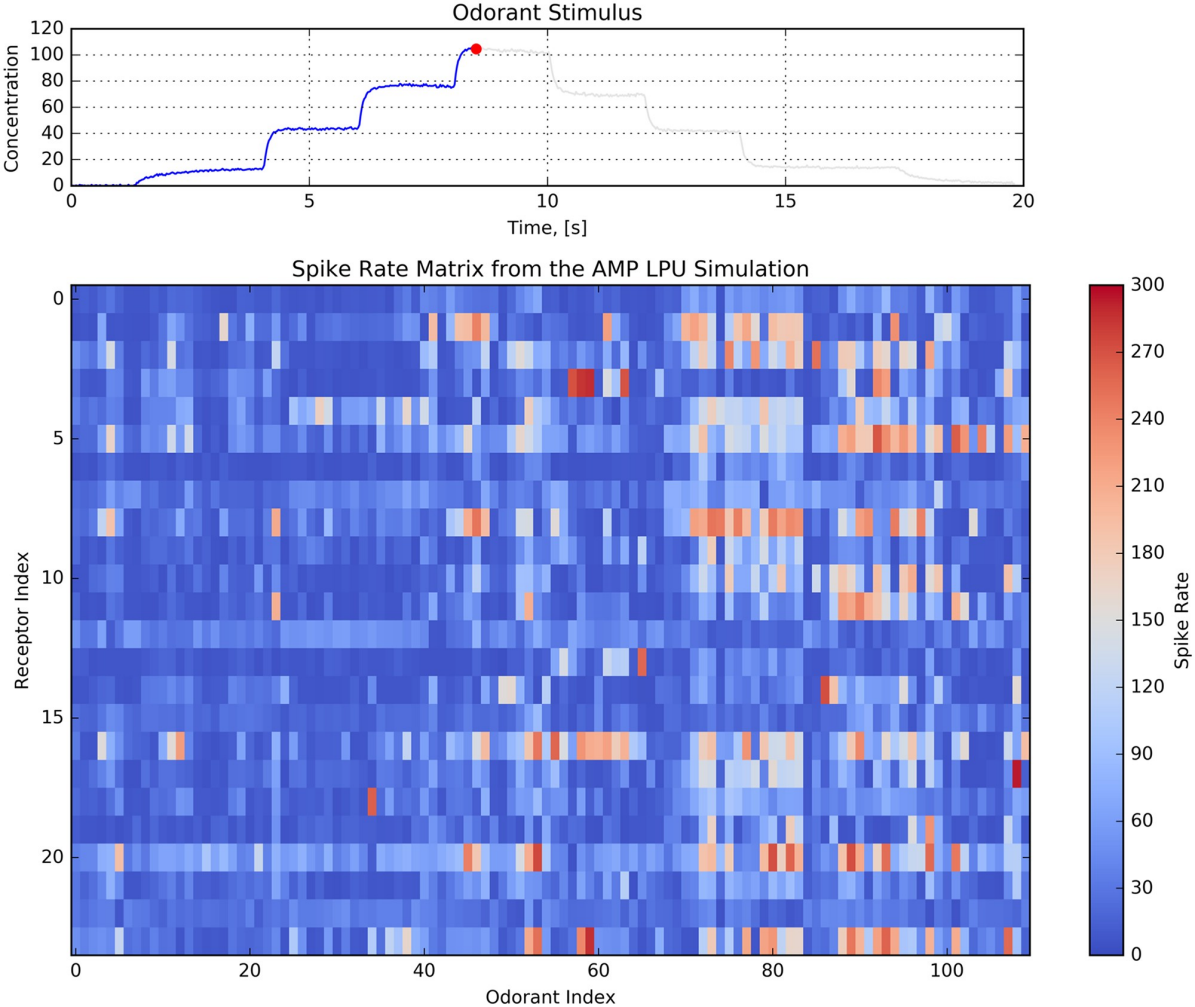
Preview of the animation of the spike rate matrix of 24 OSN groups in response to 110 odorants. Each of the OSN groups consists of 25 OTP/BSG cascades. The PSTH for each of the OSN groups is evaluated from the spike sequences generated by the 25 cascades. Each row of the matrix represents an OSN group, and each column of the matrix corresponds to an odorant. The affinity for each pairs of OSN groups and odorant is estimated using the DoOR database (see Fig 8). The dissociation of all OSN groups is assumed to be 132. (top) Staircase odorant waveform. (bottom) Dynamics of the spike rate matrix across time. See the animation in S2 Video.

To further evaluate the AMP LPU, we used the affinity matrix estimated from the DoOR database (see Fig 8), and simulated 24 OSN groups in response to 110 different odorants. The dissociation rate for OSN groups was assumed to be 132, as it can not be estimated from the available records in the DoOR database. We applied the same staircase odorant waveform as above, and visualized the PSTH of OSN groups with an animation. In Fig 10, we provide the preview and the link to the animation. As shown, the top of the animation in Fig 10 shows the staircase odorant waveform as a function of time, and the bottom of the animation (also in Fig 10) shows the spike rate matrix for 24 OSN groups and 110 odorants at each time point. Each row of the matrix represents an OSN group, and each column of the matrix corresponds to an odorant. The animation demonstrates the spatio-temporal dynamics of odorant encoding at the AMP LPU level as the intensity of the spike rate matrix varies across time. At each jump of the staircase concentration waveform, every entry in the spike rate matrix launches an upward overshoot followed by a drop down to a steady state value. Note that, the amplitude of the overshoot and the steady state values are odorant-receptor dependent.

## Discussion

Successful modeling of the encoding of odorants by olfactory sensory neurons that are spread across the antennae and maxillary palps requires an environment to easily construct and test a range of hypotheses regarding the transduction of odorants into spike trains. The essential functionality of olfactory sensory neurons that we focussed on here is their concurrent encoding of both odorant identity and odorant concentration. The resulting *concentration-dependent* spatio-temporal code determines the level of complexity of the input space driving olfactory processing in the downstream neuropils, such as odorant recognition and olfactory associative learning. It advances the state-of-the-art by creating the first explicit odorant space model and by devising a molecular spatio-temporal odorant transduction model that is extensively biologically validated.

### Odorant space

The abstract odorants (elements) of the odorant space are the tuples (**b**, **d**, *u*). Here, the binding rate tensor **b** and the dissociation rate tensor **d** model the odorant identity, while *u* = *u*(*t*), *t* ≥ 0 models the odorant concentration waveform. The binding rate tensor, the dissociation rate tensor and the concentration profile, jointly determine the level of complexity of the odorant space, and thereby, the level of complexity of the spatio-temporal encoding circuits.

### Molecular spatio-temporal odorant transduction model

We devised a class of modular OSN models as a cascade consisting of an odorant transduction process and a biophysical spike generator. The OTP model consists of an active receptor model and a co-receptor channel model. The BSG model is based on the Connor-Stevens neuron model.

The complexity of the spatio-temporal transformation in the OSNs was modeled at the molecular level. This transformation maps the odorant-receptor binding rate tensor (modulated by the odorant concentration profile) and the odorant-receptor dissociation rate tensor into OSN spike trains, respectively. The resulting *concentration-dependent spatio-temporal code* determines the level of complexity of the input space driving olfactory processing in the downstream neuropils, such as odorant recognition [45] and olfactory associative learning [46, 47].

### Biologically validated spatio-temporal odorant encoding

After developing the OTP and BSG models, we focussed on the biological validation of the spatio-temporal encoding capability of the OTP/BSG cascade. To validate our modeling approach, we examined the response of the fruit fly OSN model to odorant waveforms that were previously used in experiments with different odorants and receptors, and compared the model responses with electrophysiology recordings. By evaluating the mean squared error between the PSTH of the OTP/BSG model and the one from the experimental recording we demonstrated that the OTP/BSG cascade model closely matches the complex spatio-temporal characteristics of OSN responses at the individual level as well as at the population level of the antennae.

The output of simulated OSN population quantitatively demonstrates that the odorant identity is encoded in the set of odorant-activated OSN groups expressing the same receptor type. The spatial dimension of the spatio-temporal odorant code is provided by the number of receptor types that the OSNs express. Different odorants evoke responses in different sets of OSN groups, as shown in Fig 10. As expected, the size of the set of OSN groups expressing the same receptor type expands or reduces as the odorant concentration increases or decreases.

### Limits of biological validation

The odorant-receptor affinity matrix together with the spike rate matrix provide the macroscopic I/O characterization of the spatio-temporal encoding model at the OSN population level. However, in the absence of additional information, such as the slope, width, or peak of the OSN response to the odorant onset, the dissociation rate can not be estimated with the algorithm in Box 1 as such information is currently not available in the DoOR database. Thus, whereas previously the affinity and the dissociation rate are both estimated for multiple odorant-receptor pairs, only the affinity can be estimated for the odorant-receptor pairs recorded in the DoOR database. The dissociation rate together with the affinity are both required for reproducing the temporal response of OSNs. The latter alone can only characterize the steady state response. This illustrates some of the limitations of the DoOR datasets for characterizing the temporal response properties of OSNs, despite their richness for characterizing the steady state response of odorant-receptor pairs.

Concluding, the OSN model presented here demonstrates that the currently available data for odorant-receptor responses only enables the estimation of the affinity of the odorant-receptor pairs. Our OSN model calls for new experiments for massively identifying the odorant-receptor dissociation rates of relevance to flies.

### Future work

The OTP model was constructed upon a number of hypotheses whose validity remains to be tested. Two hypotheses are reviewed here: the modeling of the peri-receptor process as a low-pass filter and the modeling of the co-receptor channel and calcium feedback channel using variables raised to fractional power. Mechanistically, the peri-receptor process consists of a number of steps including (i) absorption of the odorant molecules through the sensillum surface, (ii) biding of odorant molecules to odorant-binding proteins (OBPs), and diffusion of bound OBPs through the sensillar lymph to OSN dendrites. The viscosity of sensillum liquor substantially reduces the molecular mobility of the odorant molecules that reach the OSN receptors. Thus the low-pass filter model of the peri-receptor process considered here. A mechanistic explanation for the fractional power of the variables describing the co-receptor channel is currently not available.

The odorant receptor and the pheromone receptor share similar temporal variability in response to input stimuli [48], despite the differences in protein structure and chemical signaling between the two receptor families. Therefore, the fruit fly OTP model can be extended to model pheromone receptors. Pheromones can be modeled as odorants with an extremely high single receptor binding and dissociation rates.

The active receptor model can be readily extended to mixtures of pure odorants. One interesting question is to study the odorant encoding of OSNs in the presence of a background odorant. Another potential direction is to investigate the “cocktail party” problem of odorant mixtures [49].

## Methods and materials

### I/O characterization of the OTP/BSG cascade

Is the OTP model given in Eq (7) capable of qualitatively reproducing transduction currents as those recorded in voltage clamp experiments? We empirically explore this question below. We set the parameters of the OTP model with the fitting result based on (*acetone*, *Or*59*b*) recordings. The binding rates and the dissociation rates of all OTP models were set to 2.17 ⋅ 10^−2^ ⋅ (*ppm* ⋅ *s*)^−1^ and 2.94 ⋅ *s*^−1^, respectively, and values of the other parameters are listed in Table 2.

**Table 2:** Summary of the parameters in the fruit fly odorant transduction model.

Variable	Value	unit	Description
*α*_1_	4.500 ⋅ 10^1^	*s*^−1^	cutoff frequency of the filter modeling the peri-receptor process
*β*_1_	8.000 ⋅ 10^−1^	unitless	slope of the transition region of the peri-receptor process filter
**γ**	2.105 ⋅ 10^−1^	*s*	scaling factor of the filtered odorant concentration gradient
*α*_2_	1.461 ⋅ 10^2^	*s*^−1^	rate of activation of the gating variable of the *co-receptor* channel
*β*_2_	1.172 ⋅ 10^2^	*s*^−1^	rate of deactivation of the gating variable of the *co-receptor* channel
*α*_3_	2.539 ⋅ 10^0^	*s*^−1^	rate of increase of the gating variable of the calcium channel
*β*_3_	9.096 ⋅ 10^−1^	*s*^−1^	rate of decrease of the gating variable of the calcium channel
*κ*	8.841 ⋅ 10^3^	*s*^−1^	feedback strength from the calcium channel to the *co-receptor* channel
*c*	6.546 ⋅ 10^−2^	unitless	value achieving the half-activation of the *co-receptor* channel
*p*	1	unitless	the Hill coefficient of the *co-receptor* channel
*I*_*max*_	6.213 ⋅ 10^1^	*pA*	maximum current amplitude generated by the *co-receptor* channel

We evaluated the model using step stimuli *u*_*s*_(*t*), ramp stimuli *u*_*r*_(*t*), and parabola stimuli *u*_*p*_(*t*), chosen as,

$$u_{s}(t)=\{\begin{array}{cc} c, & 0.5\leq t\leq 2.5\\ 0, & otherwise, \end{array}$$ (8)

$$u_{r}(t)=\{\begin{array}{cc} c\frac{1}{1.8}(t-0.5), & 0.5\leq t<2.3\\ c(1-5(t-2.3)), & 2.3\leq t<2.5\\ 0, & otherwise, \end{array}$$ (9)

$$u_{p}(t)=\{\begin{array}{cc} c{\left(\frac{1}{1.9}(t-0.5)\right)}^{2}, & 0.5\leq t<2.4\\ c{\left(1-10(t-2.4)\right)}^{2}, & 2.4\leq t<2.5\\ 0, & otherwise, \end{array}$$ (10)

where *c* is a scalar ranging between 1 and 101 with a step size of 5.

**Fig 11:**
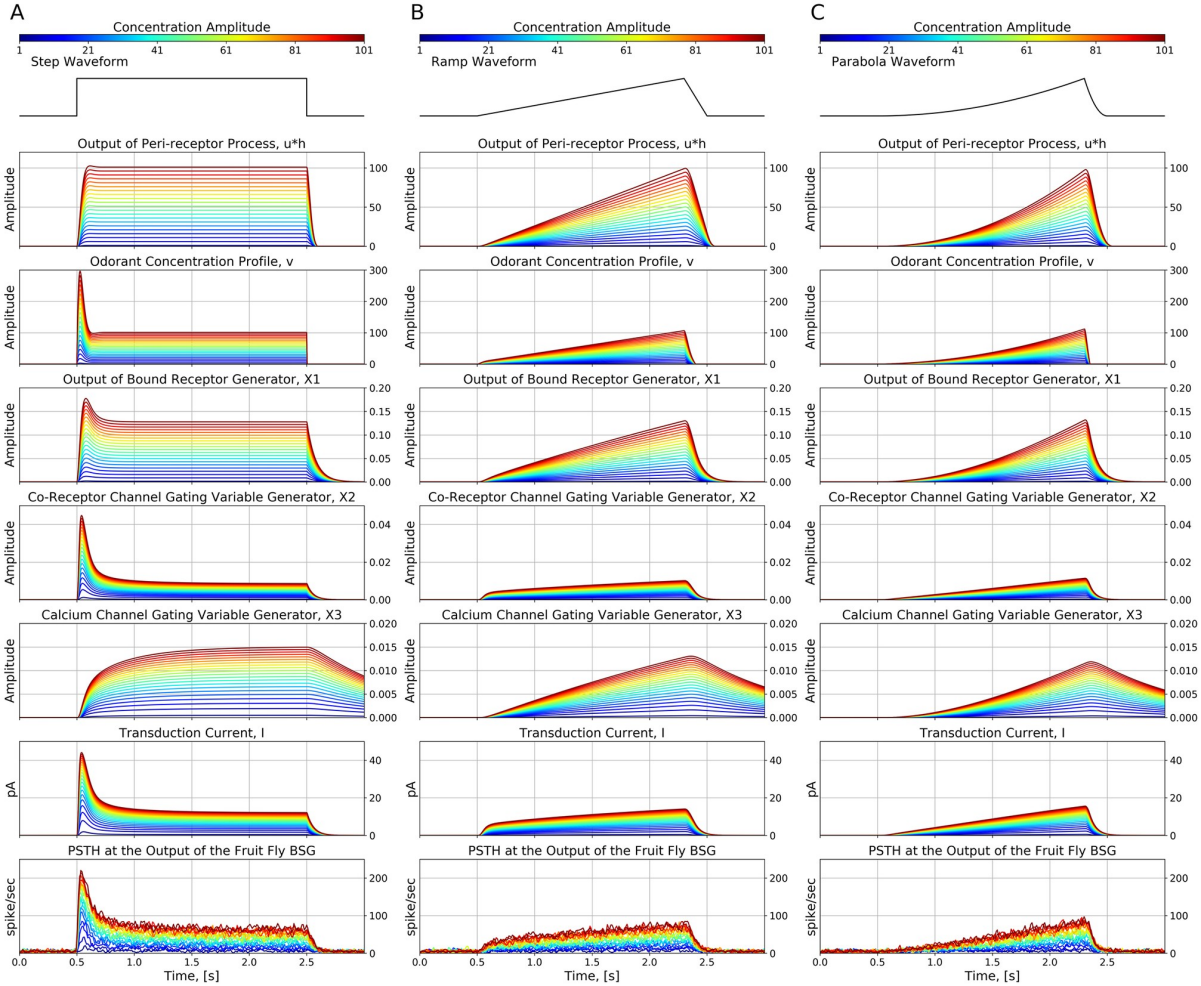
Characterization of the fruit fly OSN model in response to odorant stimuli of different concentration amplitude values. The amplitude ranges between 1 and 101 ppm with a step size of 5 ppm. The parameters of the OTP model are given in Table 2, and the parameters of the BSG model are listed in S1 Appendix. The binding rate and the dissociation rate of all OTP models were set to 2.17 ⋅ 10^−2^ ⋅ (*ppm* ⋅ *s*)^−1^ and 2.94 ⋅ *s*^−1^, respectively. (A) Step stimulus given by Eq (8). (B) Ramp stimulus given by Eq (9). (C) Parabola stimulus given by Eq (10).

The response at the output of the peri-receptor process u*h, the odorant concentration profile [**v**]_*ron*_, and the ratio of bound receptor [**x**_1_]_*ron*_ are shown in Fig 11. The slope of the rising phase of u*h after the onset of odorant is due to the effect of the filter *h*(*t*). The odorant concentration profile [**v**]_*ron*_ encodes the gradient of the concentration for the step stimuli (see the phasi-tonic response), but less so for the ramp and parabola stimuli. Lastly, the bound receptor [**x**_1_]_*ron*_ transforms the odorant concentration profile and maps it into a bounded range between 0 and 1.

As shown in Fig 11A, the OTP model exhibits temporal response dynamics akin to the adaption phenomena reported in [26]. Furthermore, we tested the OTP model with ramp and parabola stimuli of different concentration amplitude values, as shown in Fig 11.

Compared with the stimulus response of the active receptor model, the response of the OTP model exhibits a complex temporal variability, that is sensitive to both the amplitude and the gradient of the odorant stimulus waveform. For example, as shown in Fig 11B, the response of the OTP model to the ramp stimulus first reacts to the concentration gradient as the concentration amplitude is still small. After the onset of the ramp stimulus, the OTP response encodes both the concentration amplitude and the concentration gradient as it increases linearly upon a constant offset, where the linear increase represents the amplitude of the ramp stimulus and the constant offset corresponds to the constant gradient of the ramp stimulus. In addition, as shown in Fig 11C, the response of the OTP model to the parabola stimulus roughly resembles a ramp function that closely matches with the gradient of the parabola stimulus.

The complex temporal response of the OTP model is due to the feedback received by the *co-receptor channel* from the *calcium channel*. Without the *calcium channel* feedback, the OTP model is reduced to a three-stage (*peri-receptor processing*, *bound-receptor generator*, and *co-receptor channel*) feedforward model. The feedback enables the OTP model to encode the odorant concentration profile components, i.e., both the filtered odorant concentration and concentration gradient. In addition, the nonlinearities embedded in the current generation of the *co-receptor channel* (see also Eq (6)) acts as a normalization block, that facilitates the OTP model to map a stimulus with a wide range of amplitude values into a bounded transduction current.

The temporal response variability of the OTP/BSG cascade is similar to the transduction current generated by the OTP model. The similarity between the responses of the OTP model and the OTP/BSG cascade suggests that the temporal variability of the odorant concentration profile is primarily encoded in the OTP model. The BSG model is simply a sampling device mapping input current waveforms into spike trains.

### Evaluating the steady state response of the OTP/BSG cascade

We note that Eq (3) can be written as,

$$\begin{array}{c} \frac{1}{{\left[d\right]}_{ron}}\cdot \frac{d}{dt}{\left[x_{1}\right]}_{ron}=\frac{{\left[b\right]}_{ron}}{{\left[d\right]}_{ron}}\cdot {\left[v\right]}_{ron}\cdot \left(1-{\left[x_{1}\right]}_{ron}\right)-{\left[x_{1}\right]}_{ron}. \end{array}$$ (11)

where [**b**]_*ron*_/[**d**]_*ron*_ is the odorant-receptor or ligand-receptor “affinity” [50]. The active receptor model postulated in Eq (11) implies that in steady state the product between the odorant-receptor affinity and the odorant concentration profile is the main figure of merit for I/O characterization of the fruit fly OTP/BSG cascade.

To study its mapping into spike rate, we simulated OTP/BSG cascades with constant stimuli, and evaluated the spike rate at steady state.

**Fig 12:**
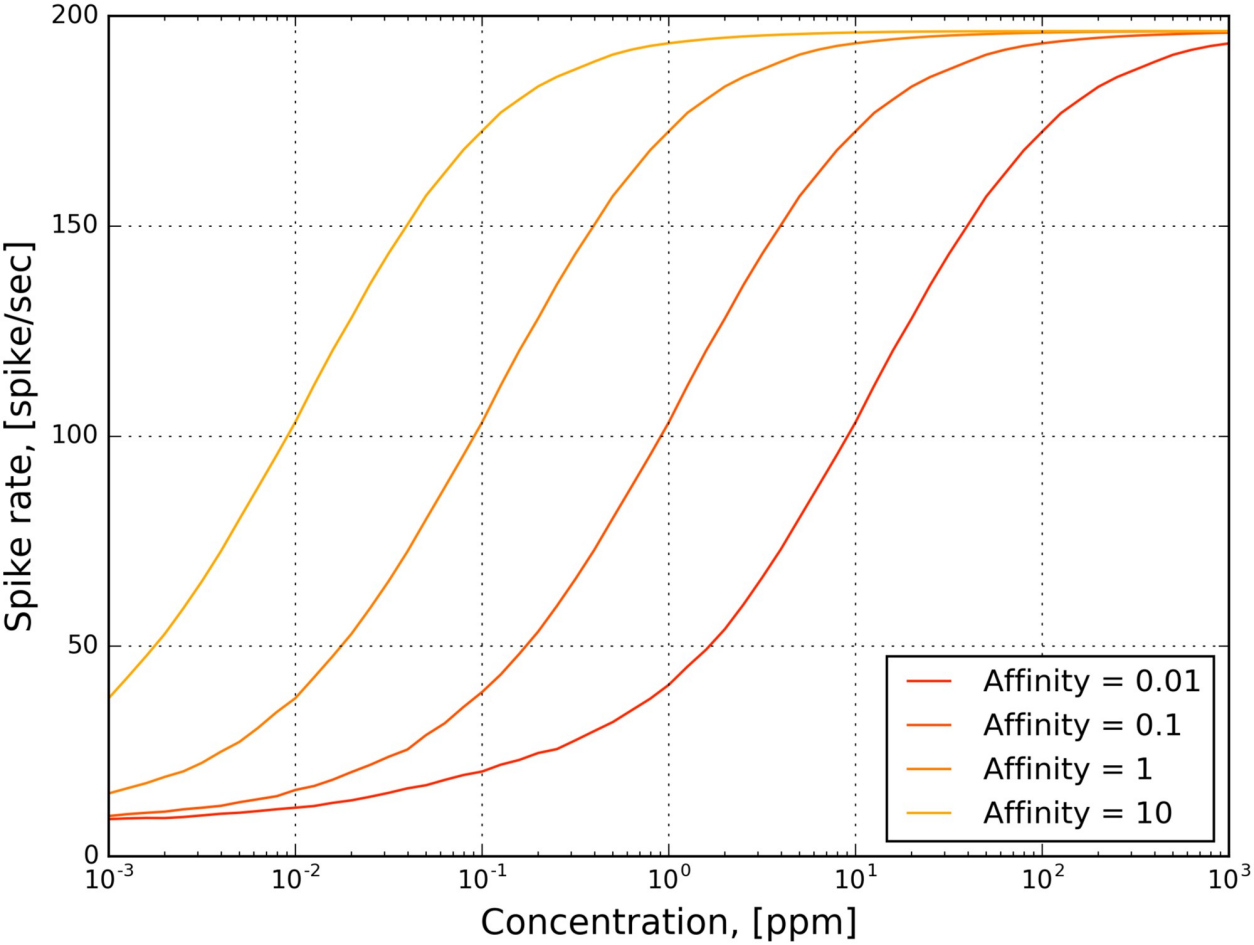
The transformation of the odorant concentration amplitude into steady-state spike rate by the OTP/BSG cascade. The transformation is characterized for fixed values of the ligand-receptor affinity in response to 5-second-long constant stimuli. The parameters of the OTP model are given in Table 2, and the parameters of the BSG model are listed in S1 Appendix. The amplitude of the constant odorant stimuli ranges between 10^−3^ and 10^3^ with a step size of 0.1 on the logarithmic scale. The spike rate is calculated in a window between 4 and 5 seconds.

The amplitude of step stimuli ranges between 10^−1^ and 10^5^ with a step size of 0.1 on the logarithmic scale. The affinity ranges between 10^−2^ and 10^1^ with a step size of 0.01 on the logarithmic scale. The parameters of the OTP model are given in Table 2, and the parameters of the BSG model are listed in S1 Appendix. The step stimulus is 5 second long, and the OTP/BSG cascades reach steady state roughly after 3 seconds. We calculated the spike rate using a window between 4 to 5 seconds, and plotted the results in 2D in Fig 12. Note that the *x*-axis in Fig 12 is on the logarithmic scale. As shown in Fig 12, for different values of the odorant-receptor affinity, the mapping of the concentration amplitude into spike rate shifts along the x-axis. A low affinity value requires a higher concentration amplitude value in order to elicit spikes above the spontaneous activity rate.

As shown in Fig 12, the transformation of the product between the odorant-receptor affinity and the concentration amplitude into spike rate resembles a sigmoidal function. The OTP/BSG cascade starts spiking only after the product exceeds a certain threshold value. For odorant-receptor pairs with a low affinity, the firing activity requires a larger minimal amplitude of concentration than for those with a higher affinity value. This, again, coincides with experimental findings that odorant-receptor pairs with lower affinity require higher odorant concentration values in order to elicit spiking activity [2].

### Reproducing the 2D encoding of the OSNs

**Fig 13:**
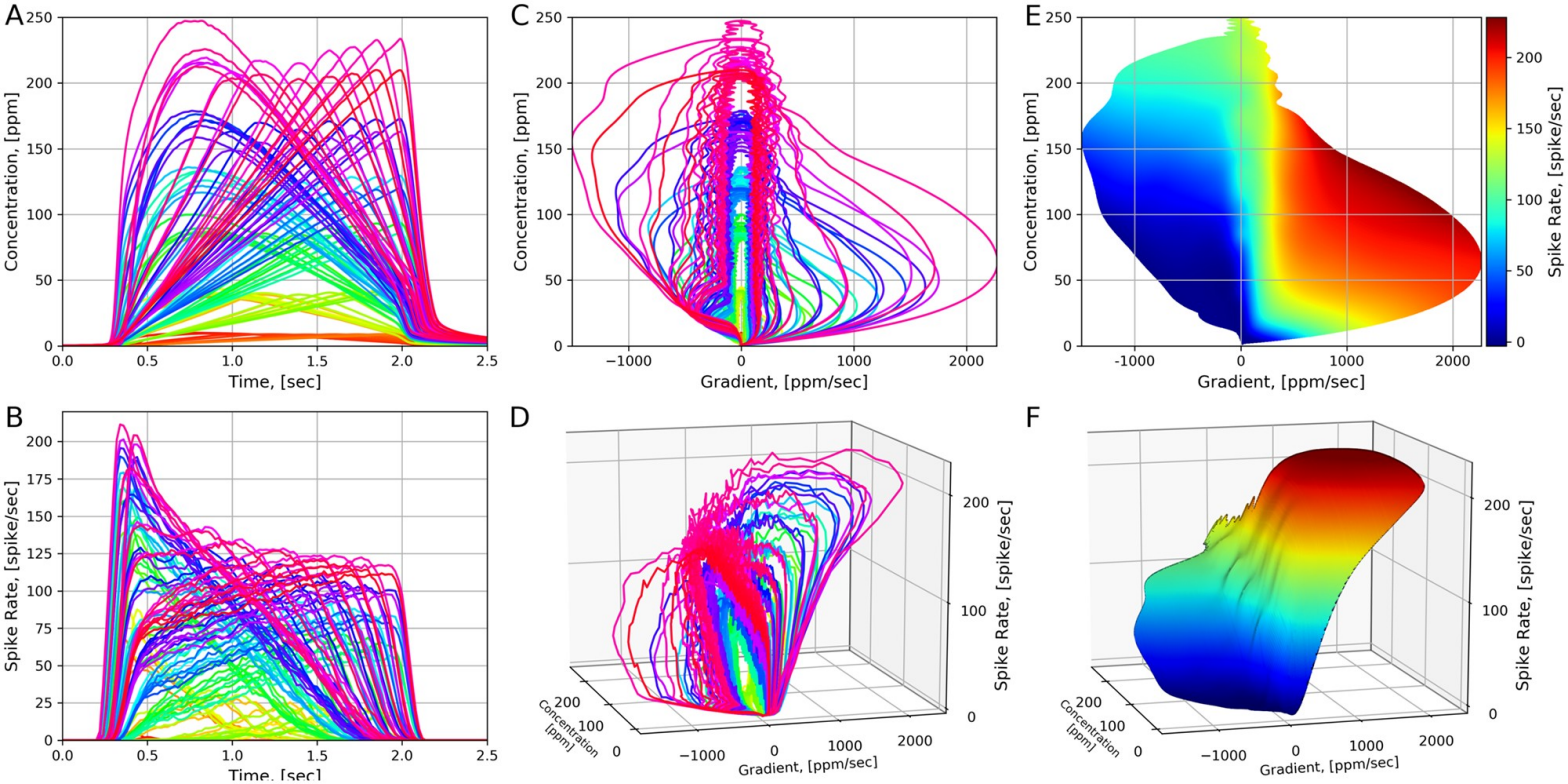
Characterizing the 2D encoding of the OTP/BSG cascade. (A) 110 triangular concentration waveforms. Different colors correspond to distinct triangular waveforms. (B) The PSTHs of the OTP/BSG cascade in response to triangular concentration waveforms. Different colors correspond to distinct waveforms. PSTHs were computed using a 20 ms bin size and a 10 ms time shift between consecutive bins. (C) The trajectories of triangular waveforms plotted in the concentration and concentration gradient plane. (D) The trajectories of PSTHs plotted in the concentration and concentration gradient plane. (E) The contour plot of the 2D manifold. (F) The 2D Encoding Manifold fitted to the trajectories of PSTHs. The manifold is generated by applying a 2D ridge estimator to the PSTHs.

To examine whether the fruit fly OTP/BSG cascade exhibits the 2D encoding property, we stimulated the cascade with the set of 110 triangular concentration waveforms that were previously used in experiments [16] with *Or59b* and *acetone*. The triangular waveforms and their trajectories are plotted in Fig 13A and 13C. We applied each of the triangular waveforms to 25 OTP/BSG cascades, and evaluated the PSTH using the spike train of all 25 cascades with a 20 ms bin size and 10 ms time shift between consecutive bins. The binding and dissociation rates of all OTP/BSG cascades was set to 2.17 ⋅ 10^−2^ ⋅ (*ppm* ⋅ *s*)^−1^ and 2.94 ⋅ *s*^−1^, respectively. The parameters of the OTP model are listed in Table 2, and the parameters of the BSG model are listed in S1 Appendix.

The responses of the OTP/BSG cascade are given in Fig 13. The PSTH of the OTP/BSG cascade in response to different waveforms is color-coded in both the 2D and 3D view, as shown in Fig 13B and 13D, respectively. In addition, we applied the 2D ridge regression algorithm to identify a 2D encoding manifold that best fits the PSTHs. The manifold and its contour are depicted in Fig 13F and 13E, respectively. Similarly to the case of the staircase waveform, the OTP/BSG cascade firing rate increases dramatically as the concentration increases.

As shown in Fig 13F, a 2D encoding manifold in a concentration and concentration gradient space provides a quantitative description of the OTP/BSG cascade. Examining Fig 13F, we note that the 2D encoding manifold is highly nonlinear and that the OTP/BSG cascade clearly encodes the odorant concentration and its rate of change. The OTP/BSG cascade responds very strongly to even the smallest positive values of the gradient and encodes only positive concentration gradients at low odorant concentrations. At high concentrations the OSN mostly encodes the odorant concentration.

### Quantitative analysis

We compared the output of the OTP/BSG model with the OSN recording data by evaluating the mean squared error (MSE) between the PSTH of the OTP/BSG model and the one from the experimental recording. We summarize in Table 3 the mean squared between the model output and the OSN recording for every odorant stimulus presented above.

**Table 3:** Mean squared error (MSE) between the model output and the OSN recording.

Stimulus	*MSE*	Odorant-Receptor pairs	Figure
Step stimuli	13.84	(*acetone*, *Or59b*)	Fig 4A
Ramp stimuli	14.92	(*acetone*, *Or59b*)	Fig 4B
Parabola stimuli	13.71	(*acetone*, *Or59b*)	Fig 4C
White noise stimulus	8.94	(*acetone*, *Or59b*)	Fig 5A
Staircase stimulus	8.85	(*acetone*, *Or59b*)	Fig 5B
Waveform 1	10.49	(*acetone*, *Or59b*)	Fig 6
Waveform 2	7.53	(*acetone*, *Or59b*)	Fig 6
Waveform 3	7.94	(*acetone*, *Or59b*)	Fig 6
Waveform 4	9.02	(*acetone*, *Or59b*)	Fig 6
Waveform 1	12.64	(*2-Butanone*, *Or59b*)	Fig 6
Waveform 2	10.40	(*2-Butanone*, *Or59b*)	Fig 6
Waveform 3	11.88	(*2-Butanone*, *Or59b*)	Fig 6
Waveform 4	10.38	(*2-Butanone*, *Or59b*)	Fig 6
Step stimuli	12.33	(*acetone*, *Or59b*)	Fig 7A
Ramp stimuli	13.12	(*acetone*, *Or59b*)	Fig 7A
Parabola stimuli	12.41	(*acetone*, *Or59b*)	Fig 7A
Step stimuli	15.61	(*methyl butyrate*, *Or59b*)	Fig 7B
Ramp stimuli	15.14	(*methyl butyrate*, *Or59b*)	Fig 7B
Parabola stimuli	14.27	(*methyl butyrate*, *Or59b*)	Fig 7B
Step stimuli	13.31	(*butyraldehyde*, *Or7a*)	Fig 7C
Ramp stimuli	12.21	(*butyraldehyde*, *Or7a*)	Fig 7C
Parabola stimuli	12.53	(*butyraldehyde*, *Or7a*)	Fig 7C

### Optimizing the OTP model

The OTP model contains 13 parameters for all odorant-receptor pairs out of which 11 are listed in Table 2 (the binding rate and the dissociation rate are not listed). We simultaneously fitted the value of every parameter by applying the “Differential Evolution” algorithm [51] to an OTP/BSG cascade with odorant concentration waveforms and OSN spike recordings. We defined loss functions $L_{i},i=1,2,3,$ as the mean squared error between the PSTH of the OSN model output and the PSTH of the OSN recordings. The Differential Evolution algorithm fitted the values of all parameters by minimizing a weighted sum of loss functions.

For (*acetone*, *Or59b*), the optimization algorithm minimizes the regularized objective function,

$$\begin{array}{c} L=L_{1}+\lambda L_{2}, \end{array}$$

where $L_{1}$ is the MSE between the PSTH of the OSN model output and the PSTH of the OSN recordings in response to the white noise waveform, and $L_{2}$ is the MSE between the PSTH of the OSN model output and the PSTH of the OSN recordings in response to the pulse-like waveforms in the Dataset 1 in S1 Appendix. Here, λ = 40 is a hyperparameter that balances the two loss functions. The fitted parameters are listed in Table 2.

To validate the optimization result for the (*acetone*, *Or59b*) pair, we applied the Different Evolution algorithm with another dataset and loss function,

$$\begin{array}{c} L=L_{1}+\lambda L_{3}, \end{array}$$ (11)

where *L*_1_ is the same as before, λ = 40 is a hyperparameter, and $L_{3}$ uses the Dataset 2 in S1 Appendix.

We initialized the Differential Evolution algorithm with a population of 5, 000 randomly generated candidate parameters. Each candidate parameter was represented as a position in the parameter space. At each iteration (evolution), the algorithm moves the candidate in the parameter space by linearly combining the parameter positions of the given population. If the new position of a candidate parameter improves the objective function, the old position is replaced by the new position. Otherwise the new position is simply discarded. We typically ran the algorithm over 10, 000 iterations.

**Fig 14:**
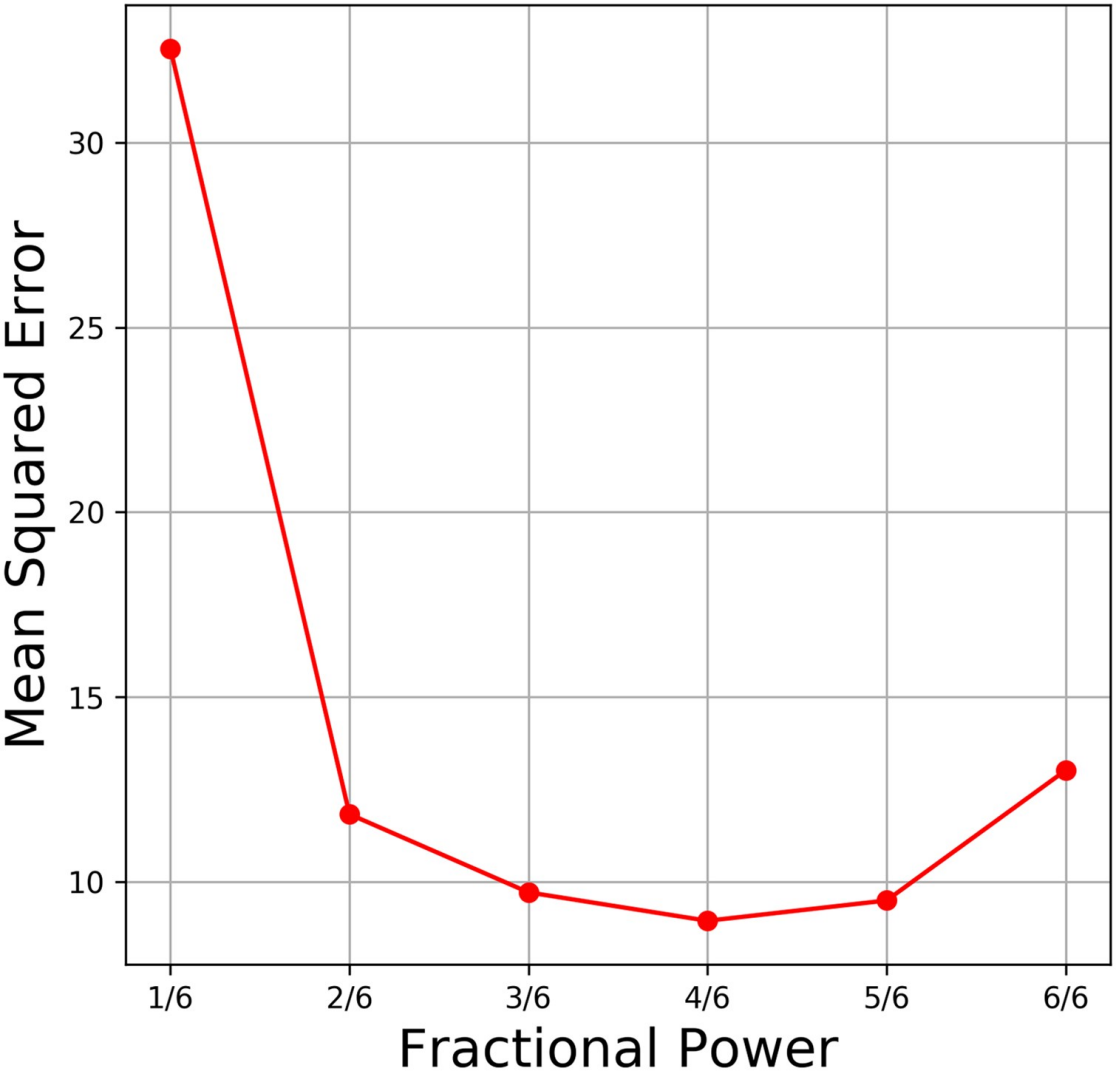
The optimal fractional power in the co-receptor channel model. We evaluated the OTP model with different fractional power values and conducted tests with white noise waveforms such as the one shown in Fig 5A. Fractional power values range between 1/6 and 1 with a gap equal to 1/6.

To obtain the optimal value of the fractional power in the co-receptor channel model, we evaluated the OTP model response. We tested the OTP model with white noise waveforms such as the one shown in Fig 5A and calculated the mean squared error between the model output and the biological OSN PSTH recording. As shown in Fig 14, the fractional power 2/3 gives the lowest mean squared error (8.94). Note that, the OTP model with the non-fractional power of 1 (proposed in [25]) results in a larger mean squared error (13.01) and is poorly reproducing the PSTH response of the biological OSN.

### Evaluating the functional significance of the odorant concentration profile

**Fig 15:**
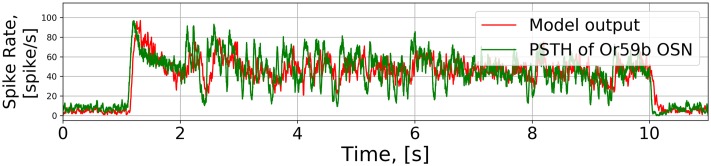
Comparison between the OSN PSTH recording and the output of the OTP/BSG model with the concentration gradient in Eq (7) set to zero. The fitted model was tested with the white noise waveform shown in Fig 5A.

To investigate the functional significance of the concentration gradient in the odorant concentration profile, we set [γ]_*ron*_ = 0 in Eq (7). As shown in Fig 15, the output of the OSN model driven with the concentration gradient set to zero reproduces the biological OSN PSTH recording poorly, with a 14.10 MSE. On the other hand, as shown in Fig 5A and Table 3, the output of the OSN model with the added term due to the concentration gradient matches closely the OSN PSTH recording with a 8.94 MSE.

### Estimation algorithm for affinity value and dissociation rate

The receptor expressed by an OSN encodes an odorant as the pair ([**b**]_*ron*_ ⋅ [**v**]_*ron*_, [**d**]_*ron*_), i.e., the product of the odorant-receptor binding rate and the odorant concentration profile, and the odorant-receptor dissociation rate. The OTP/BSG cascade then samples and presents this representation as a train of spikes. As shown in Eq (11), for a constant stimulus with amplitude *u*, the pair is equivalent to $\left(\frac{{\left[b\right]}_{ron}}{{\left[d\right]}_{ron}}\cdot {\left[v\right]}_{ron},1\right)$. In addition, we also note that [**d**]_*ron*_ ⋅ *dt* = *d*([**d**]_*ron*_ *t*) is, in effect, a time change. An algorithm to estimate the values of the odorant-receptor biding and dissociation rates may, therefore,

- estimate the ligand-receptor affinity in steady state when the left-hand-side of Eq (11) is zero for all values of the dissociation rate [**d**]_*ron*_, and
- estimate of the dissociation rate [**d**]_*ron*_ during a concentration jump assuming the value of the ligand-receptor affinity to be the one obtained in 1. above.

We describe the procedure above in more detail in Box 1.

**Box 1 Estimation algorithm of the affinity, binding and dissociation rates**

1. **procedure** given step stimulus, steady state spike rate, and peak spike rate.
2. Empirically determine the inverse mapping from spike rate to affinity.
3. Estimate the affinity value $\frac{{\left[b\right]}_{ron}}{{\left[d\right]}_{ron}}$ from the spike rate using the inverse mapping obtained under 2 above.
4. pirically determine the inverse mapping from peak spike rate to dissociation rate given the estimated affinity value $\frac{{\left[b\right]}_{ron}}{{\left[d\right]}_{ron}}$.
5. Estimate the dissociation value [**d**]_*ron*_ from the peak spike rate using the inverse mapping obtained under 4. above.
6. Compute the binding rate [**b**]_*ron*_ from the product of estimated values of affinity and dissociation rate, i.e., $\frac{{\left[b\right]}_{ron}}{{\left[d\right]}_{ron}}\cdot {\left[d\right]}_{ron}$.
7. **end procedure**

### Numerical stability of the OSN model

It is easy to see that [**x**_1_]_*ron*_ and [**x**_2_]_*ron*_ take values in [0, 1]. This is because the value of the derivative ${\left[{\dot{x}}_{1}\right]}_{ron}$ at [**x**_1_]_*ron*_ = 0 is positive and the derivative ${\left[{\dot{x}}_{1}\right]}_{ron}$ at [**x**_1_]_*ron*_ = 1 is negative. Same reasoning applies to ${\left[{\dot{x}}_{2}\right]}_{ron}$. Finally, we also note that [**x**_3_]_*ron*_ is positive.

### Simulation setup in the fruit fly brain observatory platform

The Fruit Fly Brain Observatory (FFBO) [44] is an open-source platform for the emulation and biological validation of fruit fly brain models in health and disease. It provides users highly intuitive tools to execute neural circuit models on modern commodity hardware [52]. NeuroGFX is the key FFBO component supporting the implementation of AMP LPU. NeuroGFX conjoins the simultaneous operations of model exploration and model execution with a unified graphical web interface that renders interactive simulation results.

We evaluated the response to each of the stimuli by 50 neuron groups, each group consisting of the same OSN type. Each of 50 groups consisted of 25 OSNs, and in total there were 1, 250 OSNs. We then computed the PSTH for each of OSN groups using the resultant 25 spike sequences in each of the groups. The PSTH had a 20 ms bin size and was shifted by a 10 ms time interval between consecutive bins. The parameters of all OTP models are given in Table 2. The binding rate was separately set for each odorant-receptor pair. We used the same set of parameters for all 1250 cascades, but generated different sample paths for the Brownian motion term **W** in Eq (A1) in S1 Appendix. The parameters of the BSG model are listed in S1 Appendix. A demonstration of the antenna model execution is provided in a Jupyter Notebook included in S1 Notebook.

## Supporting information

S1 Appendix (PDF)

S1 Video The animation demonstrating the AMP LPU in response to a staircase concentration waveform. (MP4)

S2 Video The animation of the spike rate matrix of 24 OSN groups in response to 110 odorants. (MP4)

S1 Notebook The Jupyter notebook in HTML format for the models and figures provided in the manuscript. (HTML)
