# Supplementary material for: A molecular odorant transduction model and the complexity of spatio-temporal encoding in the Drosophila antenna
Source: PLoS Comput Biol. 2020 Apr 14;16(4):e1007751. doi: 10.1371/journal.pcbi.1007751 (PMC7182276; doi:10.1371/journal.pcbi.1007751)
Supplement: S1 Appendix — (PDF) [file pcbi.1007751.s001.pdf]

Appendix

Peri-receptor processing model

$h(t)$ in Eq (2) is the impulse response of a low-pass linear filter that is usually defined in the literature in frequency domain. Alternatively, $h(t)$ is the solution to the second-order differential equation,

$$\frac{d^2}{dt^2}h(t) + 2\alpha_1\beta_1\frac{d}{dt}h(t) + \alpha_1^2 h(t) = \alpha_1^2\delta(t),$$

with the initial condition $h(0) = 0$ and $dh/dt|_{t=0} = 0$, where δ is the Dirac-function. The value of α_1 and β_1 are given in Table 2, and the corresponding $h(t)$ has an effective bandwidth of 45 Hz.

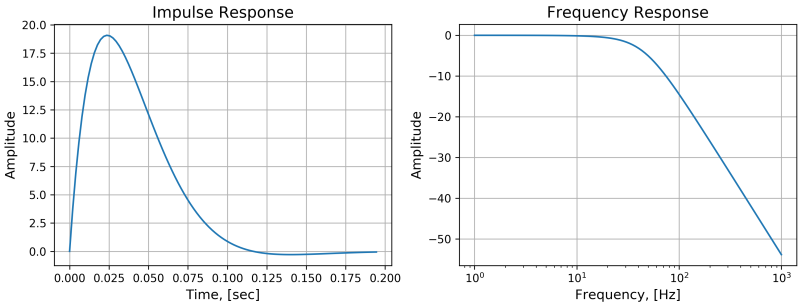

Fig A1. Impulse Response and the Frequency Response of the Peri-receptor Process. (left) The impulse response. **(right)** The frequency response.

Biophysical spike generator model

We restrict our choice of the spiking mechanism of OSNs to biophysical spike generators (BSG) such as the Hodgkin-Huxley, the Morris-Lecar, and the Connor-Stevens point neuron models. For simplicity of presentation, we only describe here the Connor-Stevens (CS) neuron model [1]. The CS model can be expressed in compact form as

$$\frac{d}{dt}[\mathbf{y}]_{ron} = \mathbf{f}([\mathbf{y}]_{ron}, [\mathbf{I}]_{ron}),$$

with $\mathbf{y} = [V, n, m, h, p, q]^T$ is a vector of state variables, $\mathbf{f}$ is a vector function of the same dimension, and $\mathbf{I}$ is the transduction current generated by the OTP model. Here ron takes the same values as the same subscript in the OTP model. Compared with the classic Hodgkin-Huxley neuron model, the CS neuron model has a continuous F-I curve [2], and is capable of encoding weak pulse stimuli with low spiking rates. It also has a wide spiking rate range that sufficiently covers the spiking rate range of the OSNs.

The CS neuron model does not fire spontaneously, and requires a minimum value of the input current to trigger firing. OSNs are noisy and fire spontaneously on average 8 spikes/s. To mitigate this mismatch, we added noise to the CS neuron model,

$$d[\mathbf{y}]_{ron} = \mathbf{f}([\mathbf{y}]_{ron}, [\mathbf{I}]_{ron})dt + d[\mathbf{W}]_{ron}, \tag{A1}$$

where $\mathbf{W} = [0, \sigma W_n, \sigma W_m, \sigma W_h, \sigma W_a, \sigma W_b]^T$, and $(W_n, W_n, W_h, W_p, W_q)$ are zero mean, unit variance independent Brownian motion processes, and σ is a scalar. We empirically determined the value of σ to be 2.05 by sweeping its value in the range of $(0, 2.5)$ so that the noisy CS model fires in average 8 spikes per second. The F-I curve of the CS neuron model for different values of σ is shown in Fig A2.

The CS model is based on the classic Hodgkin-Huxley (HH) neuron model [3] and can be expressed in compact form as

$$\frac{d}{dt}\mathbf{y} = \mathbf{f}(\mathbf{y}, I), \tag{A2}$$

where $\mathbf{y} = [V, n, m, h, p, q]^T$ is a vector of state variables, $\mathbf{f}$ is a vector function of the same dimension. For simplicity, Eq (A2) omits the subscript notation in Eq (A1). Similarly to the HH neuron model, the state variable n is a gating variable representing the activation of the potassium channel, while the state variables m and h are gating variables representing the activation and deactivation of the sodium channel, respectively. Furthermore, the variables p and q are the gating variables representing the activation and deactivation of the "a"-channel. In more detail, Eq (A2) is given by

$$\begin{aligned}
\frac{dV}{dt} &= I - I_K - I_{leak} - I_{Na} - I_a \\
\frac{dn}{dt} &= (n_\infty(V) - n)/n_\tau(V) \\
\frac{dm}{dt} &= (m_\infty(V) - m)/m_\tau(V) \\
\frac{dh}{dt} &= (h_\infty(V) - h)/h_\tau(V) \\
\frac{dp}{dt} &= (p_\infty(V) - p)/p_\tau(V) \\
\frac{dq}{dt} &= (q_\infty(V) - q)/q_\tau(V),
\end{aligned}$$

where V denotes the membrane voltage of the neuron model, and I denotes the external current. Finally,

$$
\begin{aligned}
n_\infty(V) &= \frac{\alpha_n(V)}{\alpha_n(V)+\beta_n(V)} \\
n_\tau(V) &= \frac{2}{0.38(\alpha_n(V)+\beta_n(V))} \\
m_\infty(V) &= \frac{\alpha_m(V)}{\alpha_m(V)+\beta_m(V)} \\
m_\tau(V) &= \frac{1}{0.38(\alpha_m(V)+\beta_m(V))} \\
h_\infty(V) &= \frac{\alpha_h(V)}{\alpha_h(V)+\beta_h(V)} \\
h_\tau(V) &= \frac{1}{0.38(\alpha_h(V)+\beta_h(V))} \\
p_\infty(V) &= (0.0761 \cdot \frac{\exp((V+94.22)/31.84)}{1+\exp((V+1.17)/28.93)})^{0.3333} \\
p_\tau(V) &= 0.3632 + \frac{1.158}{1+\exp((V+55.96)/20.12)} \\
q_\infty(V) &= (\frac{1}{1+\exp((V+53.3)/14.54)})^4 \\
q_\tau(V) &= 1.24 + \frac{2.678}{1+\exp((V+50)/16.027)}
\end{aligned}
$$

and,

$$
\begin{aligned}
\alpha_n(V) &= 0.01 \cdot \frac{V+55}{1-\exp(-\frac{V+55}{10})} \\
\beta_n(V) &= 0.125\exp(-\frac{V+65}{80}) \\
\alpha_m(V) &= 0.1 \cdot \frac{V+40}{1-\exp(-\frac{V+40}{10})} \\
\beta_m(V) &= 4\exp(-\frac{V+65}{18}) \\
\alpha_h(V) &= 0.7\exp(-\frac{V+65}{20}) \\
\beta_h(V) &= \frac{1}{1+\exp(-\frac{V+35}{10})}.
\end{aligned}
$$

Lastly, the current generated by each of the channels is given by,

$$
\begin{aligned}
I_K &= 20n^4(V+72) \\
I_{Na} &= 120hm^3(V-55) \\
I_{leak} &= 0.3(V+17) \\
I_a &= 47.7p^3q(V+75).
\end{aligned}
$$

Two (*acetone*, *Or59b*) datasets.

Each of the two datasets contains the PSTHs obtained from the response of OSNs expressing *Or59b* to acetone step waveforms with different concentration amplitudes.

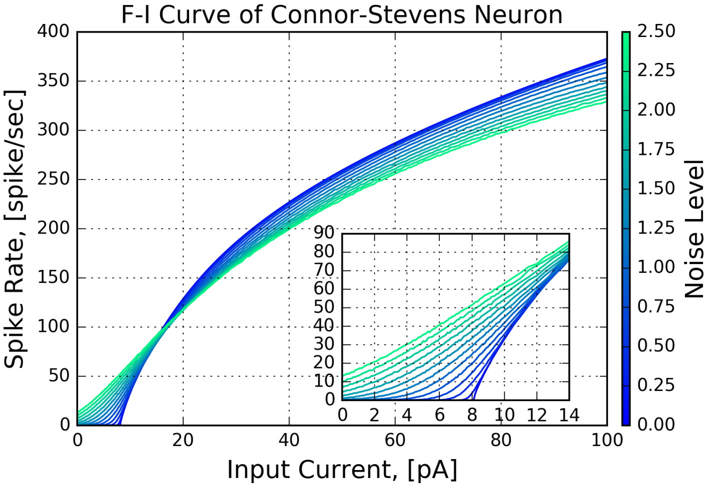

Fig A2. Characterization of the Connor-Stevens neuron model. The F-I curves of the model are color-coded for different noise levels (σ in Eq (A1)).

The peak and steady state spike rate as a function of concentration amplitude for both datasets are given in Fig A3A. The acetone step waveforms and the corresponding PSTHs of *Or59b* OSN for the two datasets are shown in Fig A3B-C.

The two datasets are part of a repository of electrophysiology recording data for the olfactory system of the fruit fly. The details of the electrophysiology recordings setup and the odorant delivery system are given in [4] and [5]. The first dataset is made public here, while the second dataset was previously published in [5]. The PSTH of the first dataset was computed using a 100 ms bin size and shifted by 25 ms between consecutive bins.

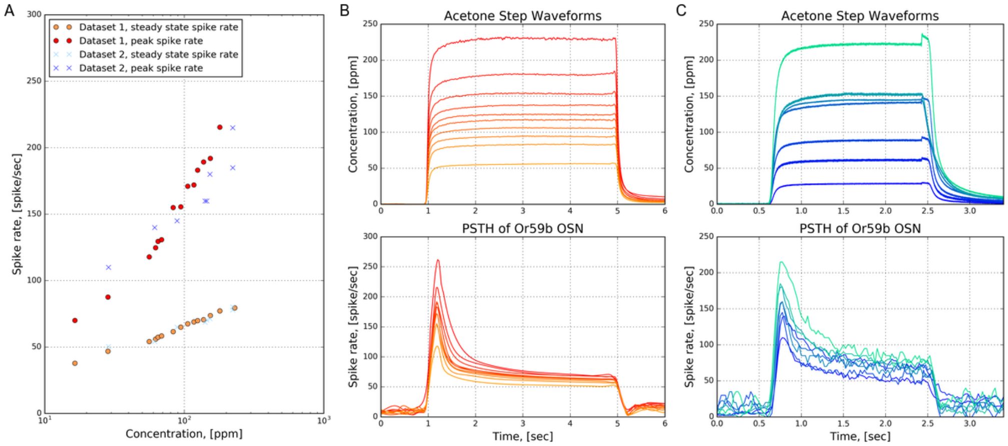

Fig A3. Two datasets of PSTHs of *Or59b*. (A) The peak and steady state spike rate as a function ofconcentration amplitude. (B) Dataset 1. (top) acetone waveforms. (bottom) PSTHs of *Or59b* OSNs. (C) Dataset 2. (top) acetone waveforms. (bottom) PSTHs of *Or59b* OSNs.

The AMP LPU in response to a staircase concentration waveform.

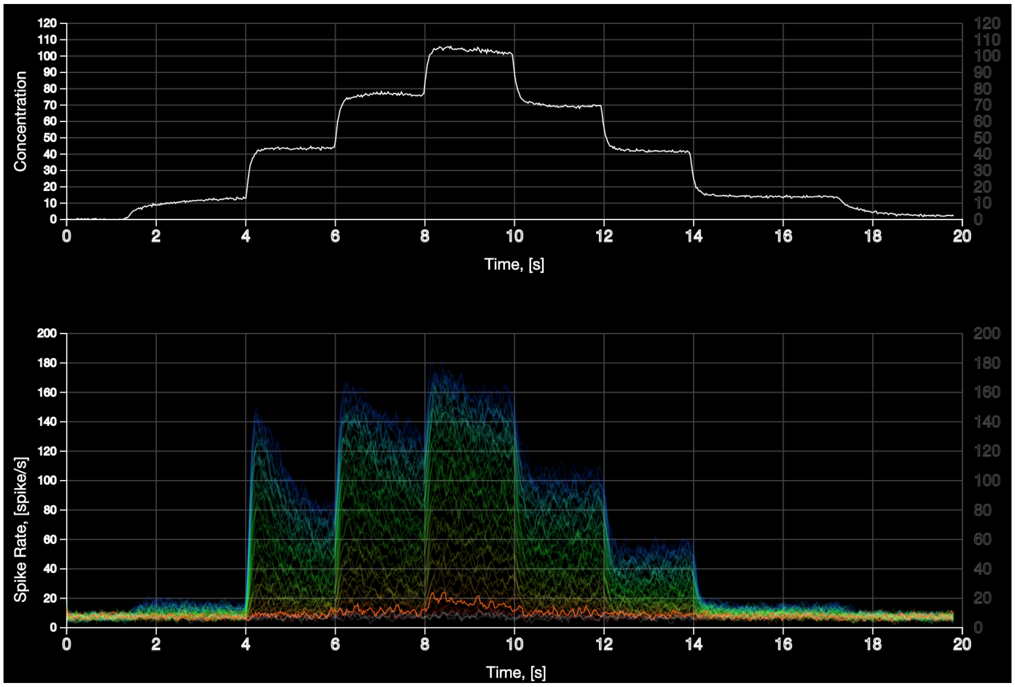

Fig A4. The 2D preview of the animation in S1 Video. (top) The staircase odorant waveform. (bottom) The PSTH of 50 receptor types, each of which is labeled with a unique color code. The orange one is highlighted.

References

1. Connor J, Stevens C. Prediction of repetitive firing behaviour from voltage clamp data on an isolated neurone soma. The Journal of Physiology. 1971;213(1):31–53.

2. Gabbiani F, Cox SJ. Mathematics for neuroscientists. Academic Press; 2010.

3. Hodgkin AL, Huxley AF. A quantitative description of membrane current and its application to conduction and excitation in nerve. The Journal of physiology. 1952;117(4):500–544.

4. Kim AJ, Lazar AA, Slutskiy YB. System identification of Drosophila olfactory sensory neurons. Journal of computational neuroscience. 2011;30(1):143–161.

5. Kim AJ, Lazar AA, Slutskiy YB. Projection neurons in Drosophila antennal lobes signal the acceleration of odor concentrations. Elife. 2015;4:e06651.
