# Supplementary material for: A molecular odorant transduction model and the complexity of spatio-temporal encoding in the Drosophila antenna
Source: PLoS Comput Biol. 2020 Apr 14;16(4):e1007751. doi: 10.1371/journal.pcbi.1007751 (PMC7182276; doi:10.1371/journal.pcbi.1007751)
Supplement: S1 Notebook — (HTML) [file pcbi.1007751.s004.html]

S1\_Notebook


# A molecular odorant transduction model and the complexity of spatio-temporal encoding in the *Drosophila* antenna¶

#### Aurel A. Lazar✉ and Chung-Heng Yeh✉¶

#### Bionet Group, Department of Electrical Engineering, Columbia University, New York, NY, USA¶

In this notebook, we provide a demonstration of the antenna model execution. We test the model with a multitude of stimuli, including step, ramp, and parabola waveforms.

#### Dependencies:¶

- numpy
- pycuda
- pyneural

Addtional dependencies:

- matplotlib
- tqdm

In [1]:

```
import matplotlib
%matplotlib inline
import matplotlib.pyplot as plt
from mpl_toolkits.axes_grid1 import make_axes_locatable
import numpy as np
import pycuda
import pycuda.autoinit
import pycuda.driver as drv
import pycuda.gpuarray as garray
import random
from tqdm import tqdm

from neural import Model
from neural.utils import compute_psth, generate_stimulus, PSTH
from neural.recorder import CUDARecorder
```

## Specify the OTP Model and the BSG Model¶

#### Odorant Transduction Process Model¶

In [2]:

```
class OTP(Model):
    Default_States = dict(
        v=(0.,0, 1e9),
        I=0.,
        uh=(0., 0., 50000.),
        duh=0.,
        x1=(0., 0., 1.),
        x2=(0., 0., 1.),
        x3=(0., 0., 1000.))
    Default_Params = dict(
        br=1.,
        dr=1.,
        gamma=0.215,
        b1=0.8,
        a1=45.,
        a2=146.1,
        b2=117.2,
        a3=2.539,
        b3=0.9096,
        kappa=8841,
        p=1.,
        c=0.06546,
        Imax=62.13)
    
    def ode(self, stimulus=0.):
        self.d_x1 = self.br*self.v*(1.-self.x1) - self.dr*self.x1
        f = np.cbrt(self.x2*self.x2) * np.cbrt(self.x3*self.x3)
        self.d_x2 = self.a2*self.x1*(1.-self.x2) - self.b2*self.x2 - self.kappa*f
        self.d_x3 = self.a3*self.x2 - self.b3*self.x3

        self.I = self.Imax * self.x2 / (self.x2 + self.c)

        self.d_uh = self.duh
        self.d_duh = -2*self.a1*self.b1*self.duh + self.a1*self.a1*(stimulus-self.uh)
        self.v = self.uh + self.gamma*self.duh
```

#### Biological Spike Generator Model¶

In [3]:

```
class NoisyConnorStevens(Model):
    Time_Scale = 1e3 # s to ms
    Default_States = dict(
        spike=0., v1=-60., v2=-60.,
        v=(-60, -80, 80),
        n=(0., 0., 1.), m=(0., 0., 1.), h=(1., 0., 1.),
        a=(1., 0., 1.), b=(1., 0., 1.),
        refactory=0.)
    Default_Params = dict(ms=-5.3, ns=-4.3, hs=-12., \
        gNa=120., gK=20., gL=0.3, ga=47.7, \
        ENa=55., EK=-72., EL=-17., Ea=-75., \
        sigma=2.05, refperiod=1.)

    def ode(self, stimulus=0.):

        alpha = np.exp(-(self.v+50.+self.ns)/10.)-1.
        if abs(alpha) <= 1e-7:
            alpha = 0.1
        else:
            alpha = -0.01*(self.v+50.+self.ns)/alpha
        beta = .125*np.exp(-(self.v+60.+self.ns)/80.)
        n_inf = alpha/(alpha+beta)
        tau_n = 2./(3.8*(alpha+beta))

        alpha = np.exp(-(self.v+35.+self.ms)/10.)-1.
        if abs(alpha) <= 1e-7:
            alpha = 1.
        else:
            alpha = -.1*(self.v+35.+self.ms)/alpha
        beta = 4.*np.exp(-(self.v+60.+self.ms)/18.)
        m_inf = alpha/(alpha+beta)
        tau_m = 1./(3.8*(alpha+beta))

        alpha = .07*np.exp(-(self.v+60.+self.hs)/20.)
        beta = 1./(1.+np.exp(-(self.v+30.+self.hs)/10.))
        h_inf = alpha/(alpha+beta)
        tau_h = 1./(3.8*(alpha+beta))

        a_inf = np.cbrt(.0761*np.exp((self.v+94.22)/31.84)/(1.+np.exp((self.v+1.17)/28.93)))
        tau_a = .3632+1.158/(1.+np.exp((self.v+55.96)/20.12))
        b_inf = np.power(1/(1+np.exp((self.v+53.3)/14.54)), 4.)
        tau_b = 1.24+2.678/(1+np.exp((self.v+50)/16.027))

        i_na = self.gNa * np.power(self.m, 3) * self.h * (self.v - self.ENa)
        i_k = self.gK * np.power(self.n, 4) * (self.v - self.EK)
        i_l = self.gL * (self.v - self.EL)
        i_a = self.ga * np.power(self.a, 3) * self.b * (self.v - self.Ea)

        self.d_v = stimulus - i_na - i_k - i_l - i_a
        self.d_n = (n_inf-self.n)/tau_n + random.gauss(0., self.sigma)
        self.d_m = (m_inf-self.m)/tau_m + random.gauss(0., self.sigma)
        self.d_h = (h_inf-self.h)/tau_h + random.gauss(0., self.sigma)
        self.d_a = (a_inf-self.a)/tau_a + random.gauss(0., self.sigma)
        self.d_b = (b_inf-self.b)/tau_b + random.gauss(0., self.sigma)

        self.d_refactory = (self.refactory < 0)

    def post(self):
        self.spike = (self.v1 <= self.v2) * (self.v <= self.v2) * (self.v2 > -30.)
        self.v1 = self.v2
        self.v2 = self.v
        self.spike = (self.spike > 0.) * (self.refactory >= 0)
        self.refactory -= (self.spike > 0.) * self.refperiod
```

#### Basic Configuration¶

In [4]:

```
dtype = np.float64
dt  = 3e-5
dur = 3.

or_num = 50
osn_num = 50
tot_num = or_num * osn_num
```

#### Generate Step Waweform¶

In [5]:

```
waveform_step = generate_stimulus('step', dt, dur-dt/2, (0.5, 2.5), 100.)
t = np.arange(0, len(waveform_step)*dt-dt/2, dt)
```

#### Instantiate Models¶

In [ ]:

```
br = np.repeat(0.02*np.linspace(0.01, 1., or_num, dtype=dtype), osn_num)
dr = 10
otp = OTP(br=br, dr=dr)
bsg = NoisyConnorStevens(sigma=0.0019/np.sqrt(dt))

otp.compile(backend='cuda', num=tot_num, dtype=dtype, stimulus=0.)
bsg.compile(backend='cuda', num=tot_num, dtype=dtype)

# keep track of simulation progress
recorder_otp = CUDARecorder(otp, ['I','x2', 'x3', 'x1', 'v', 'uh'], len(t), gpu_buffer=500, callback=True)
recorder_bsg = CUDARecorder(bsg, ['spike'], len(t), gpu_buffer=500, callback=True)
```

#### Execute Models¶

In [ ]:

```
for w in tqdm(waveform_step):
    otp.update(dt, stimulus=w)
    bsg.update(dt, stimulus=otp.I)
```

#### Compute PSTH¶

In [8]:

```
psth_step = PSTH(recorder_bsg.spike[:osn_num], dt, window=20e-3, shift=10e-3)
for i in range(1, or_num):
    psth_step.merge(PSTH(recorder_bsg.spike[i*osn_num:(i+1)*osn_num], dt, 20e-3, 10e-3))
```

#### Plot Result¶

In [9]:

```
cmap = matplotlib.cm.jet
fs = 18
tkfs = 14
c = lambda x: cmap(float(x)/or_num)

fig, axes = plt.subplots(8, 1, figsize=(8,27), dpi=300)

ylabels = (
    'Amplitude',
    'Amplitude',
    'Amplitude',
    'Amplitude',
    'Amplitude',
    'pA',
    'spike/sec')
titles = (
    'Output of Peri-receptor Process, u*h',
    'Odorant Concentration Profile, v',
    'Output of Bound Receptor Generator, X1',
    'Co-Receptor Channel Gating Variable Generator, X2',
    'Calcium Channel Gating Variable Generator, X3',
    'Transduction Current, I',
    'PSTH at the Output of the BSG')

axes[7].set_xlabel('Time, [s]',fontsize=fs)

for ax in axes[1:]:
    ax.grid()
for ax, label, title in zip(axes[1:], ylabels, titles):
    ax.set_title(title, fontsize=fs)
    ax.set_ylabel(label, fontsize=fs)
    ax.yaxis.tick_right()
    ax.tick_params(axis='both', which='major', labelsize=tkfs)
    ax.tick_params(axis='both', which='minor', labelsize=tkfs)
    ax.set_xlim([t[0], t[-1]])
for ax in axes[1:-1]:
    ax.get_xaxis().set_ticklabels([])

for i,x in enumerate((
    recorder_otp['uh'][::osn_num],
    recorder_otp['v'][::osn_num],
    recorder_otp['x1'][::osn_num],
    recorder_otp['x2'][::osn_num],
    recorder_otp['x3'][::osn_num],
    recorder_otp['I'][::osn_num],
    psth_step.psth)):
    tt = t[::int(np.ceil(len(t)/x.shape[1]))]
    for j,y in enumerate(x):
        color = 'k' if i < 2 else c(j)
        axes[i+1].plot(tt,y,color=color)

axes[0].plot(t, 0.3*waveform_step/np.max(waveform_step), '-k')
axes[0].set_axis_off()
axes[0].axis([t[0], t[-1], -0.1, 1.])
axes[0].annotate('Step Waveform; 100 ppm', xy=(0.1, 0.4), fontsize=fs)

divider = make_axes_locatable(axes[0])
cax = divider.new_vertical(size="5%", pad=-1., pack_start=False)
fig.add_axes(cax)

norm = matplotlib.colors.Normalize(vmin=np.min(br), vmax=np.max(br))
cb1 = matplotlib.colorbar.ColorbarBase(cax, cmap=cmap,
                                norm=norm,
                                orientation='horizontal')
cb1.ax.xaxis.set_ticks_position('bottom')
cb1.ax.xaxis.set_label_position('top')
cb1.set_label('Binding Rate',fontsize=fs)
cax.tick_params(axis='both', which='major', labelsize=tkfs)
cax.tick_params(axis='both', which='minor', labelsize=tkfs)

plt.tight_layout()
```
